# Structure-Activity Relationship and *in Vivo* Anti-Tumor Evaluations of Dictyoceratin-A and -C, Hypoxia-Selective Growth Inhibitors from Marine Sponge

**DOI:** 10.3390/md13127074

**Published:** 2015-12-16

**Authors:** Yuji Sumii, Naoyuki Kotoku, Akinori Fukuda, Takashi Kawachi, Masayoshi Arai, Motomasa Kobayashi

**Affiliations:** Graduate School of Pharmaceutical Sciences, Osaka University, 1-6 Yamada-oka, Suita, Osaka 565-0871, Japan; y-sumii@phs.osaka-u.ac.jp (Y.S.); sukoshi1293fushigi@yahoo.co.jp (A.F.); pabro-ai@hotmail.co.jp (T.K.); araim@phs.osaka-u.ac.jp (M.A.)

**Keywords:** dictyoceratin-C, dictyoceratin-A, hypoxia-selective growth inhibitor, marine sponge, analog synthesis, structure-activity relationship, *in vivo* antitumor effect

## Abstract

Oral dictyoceratin-C (**1**) and A (**2**), hypoxia-selective growth inhibitors, showed potent *in vivo* antitumor effects in mice subcutaneously inoculated with sarcoma S180 cells. Structurally modified analogs were synthesized to assess the structure–activity relationship of the natural compounds **1** and **2** isolated from a marine sponge. Biological evaluation of these analogs showed that the exo-olefin and hydroxyl and methyl ester moieties were important for the hypoxia-selective growth inhibitory activities of **1** and **2**. Thus far, only substitution of the methyl ester with propargyl amide in **1** was found to be effective for the synthesis of probe molecules for target identification.

## 1. Introduction

Marine natural products have attracted attention in recent years as a rich and promising source of drug candidates, especially in the field of anticancer drug discovery [1]. In the majority of cases, only small quantities of the compounds could be isolated from the extracts of marine organisms such as sponges and tunicates; therefore, sustainable supply is the major limitation for further evaluation of these compounds and drug development. One of the solutions to address this issue is chemical synthesis of the active compounds and their analogs. Structure-activity relationship studies and syntheses of the truncated natural products give us further opportunities to generate more promising drug leads with optimized activity, chemical stability, and accessibility [2,3].

It is widely accepted that hypoxia aggravates tumorigenesis by promoting tumor growth, angiogenesis, and metastasis, or by inducing resistance to chemotherapy and irradiation [4]. Therefore, compounds exhibiting hypoxia-selective growth inhibitory activity could be novel and promising drug leads for anticancer drug development [5], and the adaptation factors of tumor cells to hypoxia environment, with particular regard to hypoxia inducible factor-1 (HIF-1), have been extensively investigated as drug targets for cancer chemotherapy.

**Figure 1:**
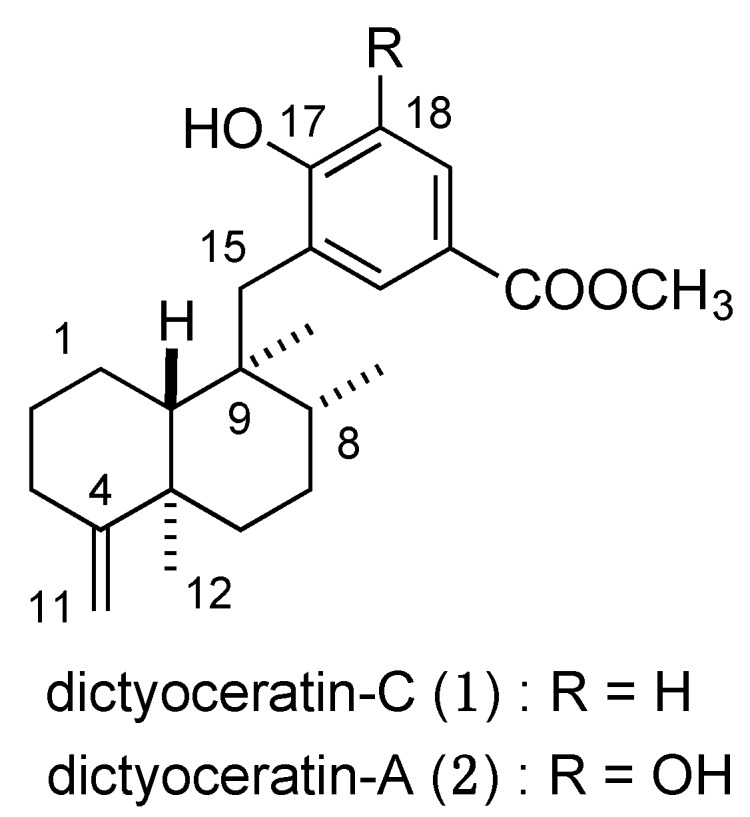
Chemical structures of (+)-dictyoceratin-C (**1**) and -A (**2**).

In our continuing search for bioactive compounds from marine organisms, we isolated dictyoceratin-C (**1**) [6] from the Indonesian marine sponge *Dactylospongia elegans* as a hypoxia-selective growth inhibitor, and found that dictyoceratin-A (**2**) [7] exhibited a similar biological activity (Figure 1). These two sesquiterpene phenols inhibited the proliferation of human prostate cancer DU145 cells selectively under hypoxic condition in a dose-dependent manner at concentrations ranging from 1.0 to 10 μM, by inhibiting the accumulation of HIF-1α under hypoxic condition [8].

To obtain compounds in sufficient yields for further evaluation, we recently reported the enantioselective total synthesis of **1** and **2**, with confirmation of absolute stereochemistry [9]. Moreover, we found that unnatural enantiomers of **1** and **2** also showed similar hypoxia-selective growth inhibitory activity against DU145 cells. It implies that the pharmacophore of these compounds is the *para*-hydroxybenzoyl ester moiety of the side chain and the chiral decalin moiety might not play a crucial role [9]. However, more precise structure-activity relationship (SAR) information is needed to develop promising anticancer drug leads based on these compounds. In this study, we investigated the *in vivo* antitumor effect of compounds **1** and **2** and analyzed their SAR through design and synthesis of various analog compounds.

## 2. Results and Discussion

### 2.1. In Vivo Antitumor Activity of **1** and **2**

Enantioselective total synthesis of **1** and **2** yielded hundreds of milligrams of these compounds [9]. In order to verify the potential of these compounds as promising drug leads for cancer treatment, we examined their *in vivo* antitumor activity in mice subcutaneously inoculated with sarcoma S180 cells.

**Figure 2:**
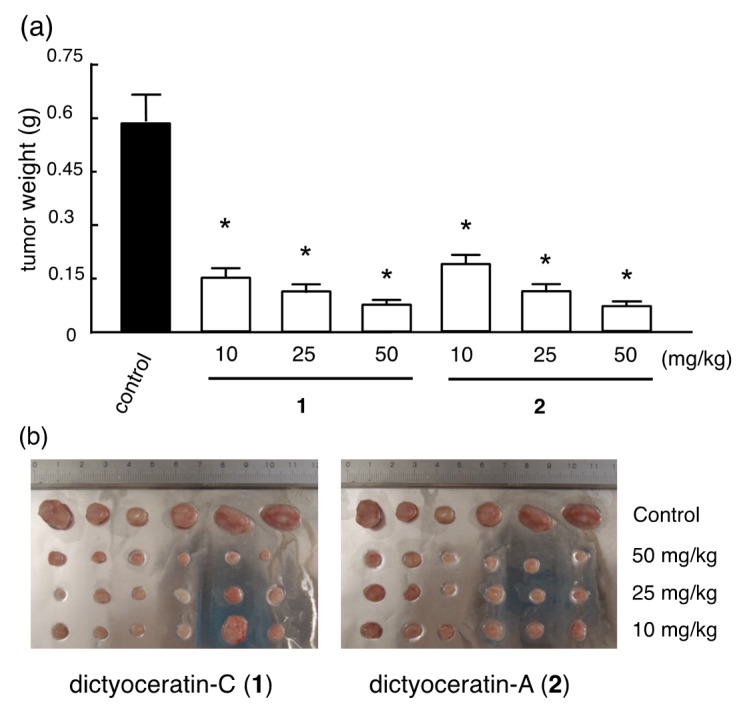
*In* *vivo* antitumor effect of dictyoceratin-C (**1**) and A (**2**). (**a**) Mean ± SD of tumor weight of each group. * *p* < 0.05; (**b**) Images of surged tumors after two weeks.

The compounds were orally administered every other day for two weeks, and the effectiveness of the compounds was determined by weighing the surged tumor on the day after final administration. Both compounds at 10–50 mg/kg inhibited the growth of implanted tumors, with ~90% reduction of the tumor weight at 50 mg/kg relative to respective control (Figure 2). Moreover, no significant acute toxicities, such as weight loss or diarrhea, were observed during the study period for both compounds. This result indicated that compounds **1** and **2** are potential anticancer drug leads.

### 2.2. Design and Synthesis of Structure-Modified Analogs

**Figure 3:**
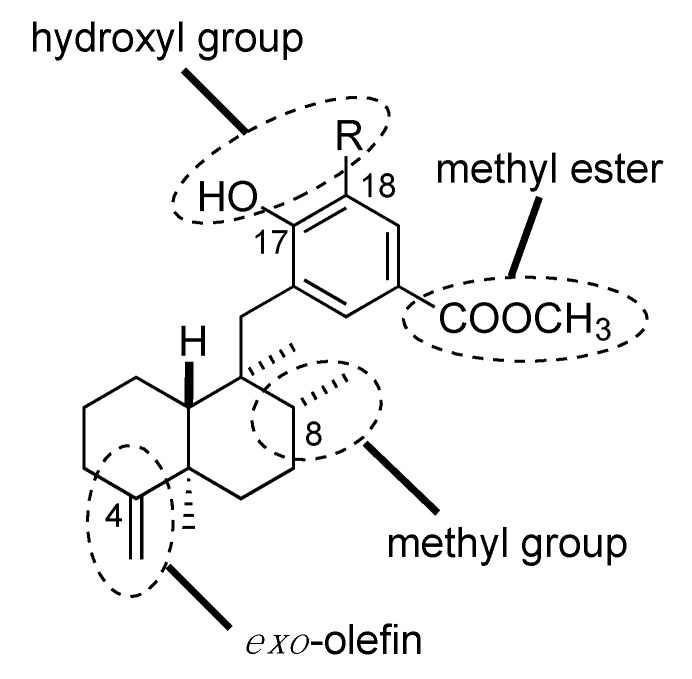
Modification of functional groups for structure-activity relationship (SAR).

SAR study of **1** or **2** was performed to identify the important moiety for their biological activities. In the initial SAR study of some natural sesquiterpene phenols/quinones isolated from sponge extracts, *para*-hydroxybenzoyl ester moiety was found to play a significant role in the hypoxia-selective growth inhibitory activity [8,9]. In order to examine the SAR in detail, we synthesized analogs of **1** or **2** by focusing on certain particular functional groups, such as methyl ester or hydroxyl group of the aromatic ring and *exo*-olefin or C-8 methyl groups in the decalin part (Figure 3).

#### 2.2.1. Modification of Aromatic Ring

**Scheme 1:**
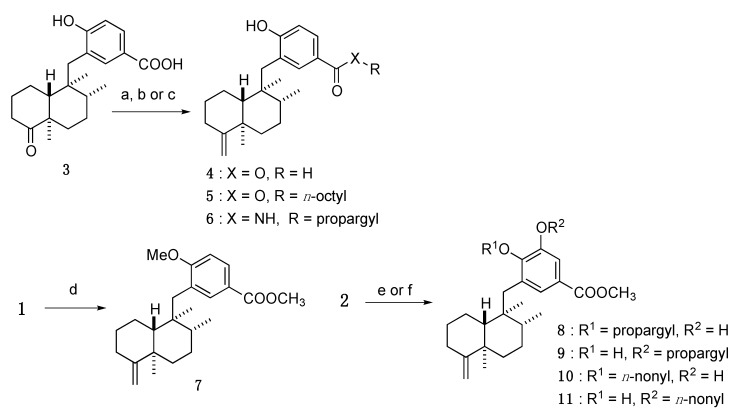
Synthesis of aromatic ring-modified analogs. Reagents and conditions: (**a**) Ph_3_PCH_3_Br, KHMDS, THF, 94%; (**b**) SOCl_2_, octanol, 50 °C, 13%; (**c**) EDCI·HCl, HOBt, propargylamine, DMF, 58%; (**d**) MeI, K_2_CO_3_, DMF, quant.; (**e**) propargyl bromide, K_2_CO_3_, DMF, **8**: 35%, **9**: 13%; (**f**) *n*-nonyl bromide, K_2_CO_3_, DMF, **10**: 21%, **11**: 14%.

Involvement of the functionality of the aromatic ring was analyzed. To elucidate the functional group crucial for the bioactivity and to develop more potent compounds, modification of the ester group and hydroxyl group of **1** was executed (Scheme 1).

First, octyl ester analog **5** and propargyl amide analog **6** were synthesized from compound **3**, an intermediate in the total synthesis of **1** [9], through Wittig olefination and subsequent condensation with *n*-octanol or propargylamine, respectively. Further, the phenolic hydroxyl group of **1** was alkylated with MeI and K_2_CO_3_ to provide a methyl ether analog **7**.

Second, participation of the respective hydroxyl groups of **2** was examined. In this analysis, propargyl and nonyl ether were selected to determine the relationship between lipophilicity/steric bulkiness and bioactivity. Alkylation of the diol group of **2** via a general method (alkyl bromide and K_2_CO_3_ in DMF) yielded a mixture of two mono-alkylated compounds, together with di-alkylated compound as a major product; a selective mono-alkylation condition could not be achieved. Fortunately, each mono-alkylated compound could be separated by open column chromatography to yield 17-*O*-alkylated analogs (**8**,**10**) and 18-*O*-alkylated analogs (**9**,**11**), respectively.

#### 2.2.2. Modification of Decalin Part

Next, modification of the decalin part was examined. Many reaction steps were needed to elaborate the decalin part in a chemo- and stereoselective manner, mainly for the construction of 8α-methyl group and relatively unstable *exo*-olefin at C-4. Therefore, synthetic study of the truncated natural compounds might be effective for obtaining analogs with improved accessibility or stability or both.

**Scheme 2:**
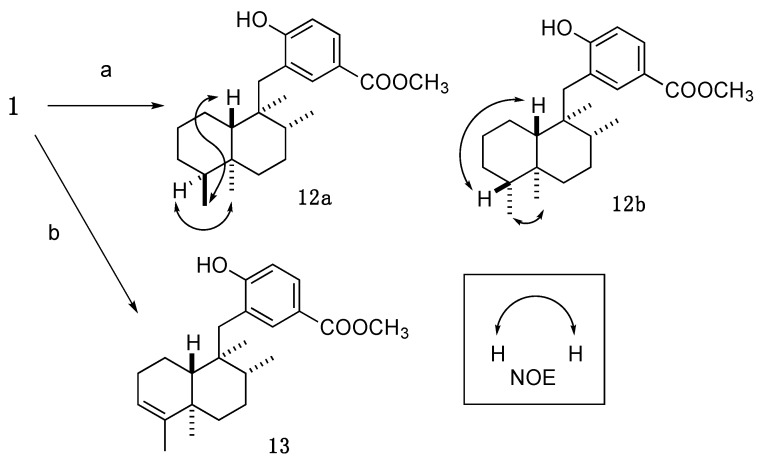
Synthesis of exo-methylene-modified analogs. Reagents and conditions: (**a**) H_2_, Pd-C, MeOH (**12a**: 51%, **12b**: 47%); (**b**) RhCl_3_·H_2_O, EtOH, reflux, 61%.

First, modifications of the *exo*-olefin at C-4 of **1** were attempted (Scheme 2). Hydrogenation of **1** yielded 3,4-dihydro analog (**12**) as a diastereomixture (1:1), which was separated by HPLC to provide **12a** (4*S*) and **12b** (4*R*). Stereochemistry of each compound was determined by NOE experiment. Isomerization of the *exo*-olefin of **1** was accomplished by treatment with RhCl_3_·H_2_O in refluxing ethanol [10] to yield compound **13** having an internal olefin.

**Scheme 3:**
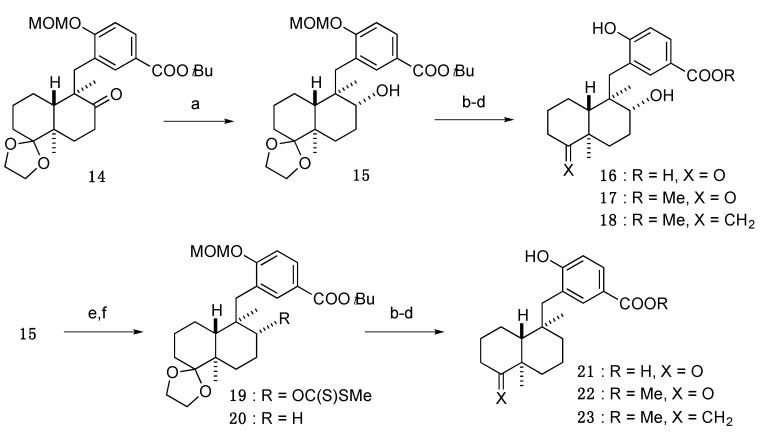
Synthesis of 8-methyl group-modified analogs. Reagents and conditions: (**a**) NaBH_4_, CeCl_3_·7H_2_O, MeOH, 94%; (**b**) 80% TFA, THF, 50 °C, **16**: 97%, **21**: 93%; (**c**) SOCl_2_, MeOH, 50 °C, **17**: 98%, **22**: 87%; (**d**) Ph_3_PCH_3_Br, KHMDS, THF, **18**: 39%, **23**: 95%; (**e**) NaH, CS_2_, THF, rt, then MeI, 50 °C, 94%; (**f**) *n*Bu_3_SnH, AIBN, toluene, 80 °C, 86%.

Next, transformation of the 8-methyl group of **1** was also examined (Scheme 3). We designed and synthesized 8-hydroxyl and 8-desmethyl analogs from 8-keto compound **14**, an intermediate in the total synthesis of **1**. Reduction of the ketone of **14** by NaBH_4_ yielded compound **15** as a single diastereomer. Multiplicity of the newly formed oxymethine proton signal in the ^1^H-NMR spectrum of **15** was ddd (*J* = 10.3, 5.2, 3.4 Hz), indicating that the reduction proceeded selectively from the β-side of the molecule. Removal of all protecting groups of **15** yielded a benzoic acid **16**, and subsequent treatment with SOCl_2_ in MeOH followed by Wittig olefination provided an 8-hydroxy analog **18**. Conversely, removal of the hydroxyl group of **15** was achieved with the *Barton–McCombie* deoxygenation reaction [11] to yield **20**, which was converted in the same manner as analog **18** to yield an 8-desmethyl analog **23**.

### 2.3. Biological Evaluation of Synthetic Analogs

**Figure 4:**
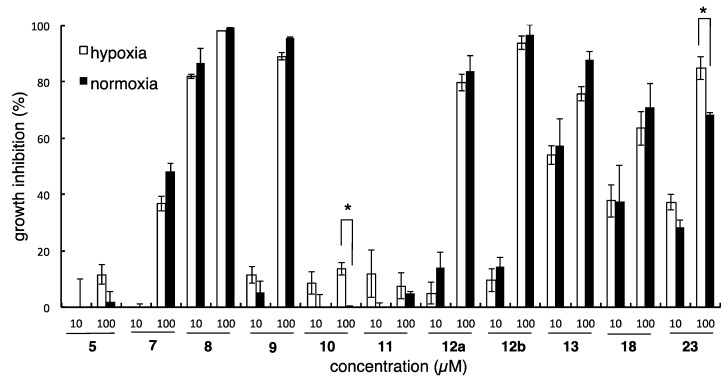
Growth inhibitory activity of dictyoceratin analogs against DU145 cells. * *p <* 0.05.

**Figure 5:**
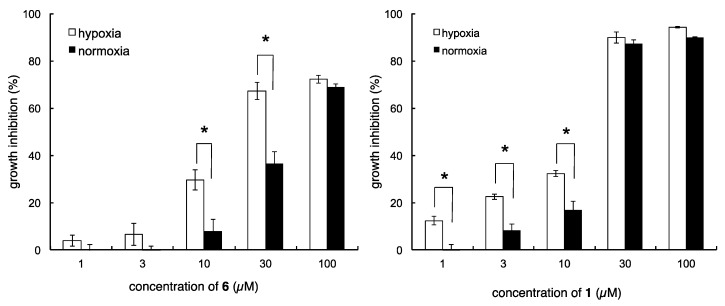
Growth inhibitory activity of dictyoceratin-C (**1**) and propargyl amide analog (**6**) against DU145 cells. * *p <* 0.05.

Following the synthesis of the desired analogs, growth inhibitory activities of the analogs against DU145 cells were evaluated under normoxic and hypoxic conditions. As briefly summarized in Figure 4, the majority of the analogs lost the hypoxia-selective growth inhibitory activity of the parent natural product. Thus, methyl ether analog **7**, 17-*O*-propargyl analog **8**, and 18-*O*-propargyl analog **9** did not possess hypoxia-selectivity. These results suggested that the 17-hydroxyl group might be necessary for hypoxia selectivity and the 18-hydroxyl group was partially concerned with growth inhibitory activity. In contrast, octyl ester analog **5**, 17-*O*-nonyl ether analog **10**, and 18-*O*-nonyl analog **11** showed only weak hypoxia-selective growth inhibitory activity. Because these analogs were highly lipophilic, this significant change in their physical properties might affect their biological activity. Furthermore, all the *exo*-olefin-modified analogs (**12a**,**12b**,**13**) and 8-hydroxyl analog (**18**) lost hypoxic/normoxic selectivity, while 8-desmethyl analog **23** showed moderate hypoxia-selective growth inhibitory activity. These results indicated that the *exo*-olefin might be an essential moiety for hypoxia-selective growth inhibitory activity and the 8-methyl group also affected growth inhibition. Taken together, not only the *para*-hydroxybenzoyl moiety but also the decalin part, including 4-*exo*-olefin and 8-methyl groups, were essential for the hypoxia-selective growth inhibitory activity of **1** and **2**. Of the synthesized analogs, only the propargyl amide analog **6** showed excellent hypoxia-selective growth inhibitory activity. In particular, potent and hypoxia-selective growth inhibitory activity was observed at 30 µM relative to the parent compound (Figure 5).

## 3. Experimental Section

### 3.1. General

The following instruments were used to obtain physical data: a JASCO P-2200 digital polarimeter (L = 50 mm) for specific rotations; a JEOL ECS-300 (^1^H-NMR: 300 MHz, ^13^C-NMR: 75 MHz), ECA-500 (^1^H-NMR: 500 MHz, ^13^C-NMR: 125 MHz) and a Varian NMR system (^1^H-NMR: 600 MHz, ^13^C-NMR: 150 MHz) spectrometer for ^1^H and ^13^C NMR data using tetramethylsilane or residual solvent peak as internal standards; a JASCO FT/IR-5300 infrared spectrometer for IR spectra; a Waters Q-Tof Ultima API mass spectrometer for ESI-TOF MS; HPLC was performed using a Hitachi L-6000 pump equipped with Hitachi L-4000H UV detector. Silica gel (Kanto, 40–100 μm) and pre-coated thin layer chromatography (TLC) plates (Merck, 60F_254_) were used for column chromatography and TLC. Spots on TLC plates were detected by spraying acidic *p*-anisaldehyde solution (*p*-anisaldehyde: 25 mL, *c*-H_2_SO_4_: 25 mL, AcOH: 5 mL, EtOH: 425 mL) or phosphomolybdic acid solution (phosphomolybdic acid: 25 g, EtOH: 500 mL) with subsequent heating. Unless otherwise noted, all the reaction was performed under N_2_ atmosphere. After workup, the organic layer was dried over Na_2_SO_4_. ^1^H and ^13^C NMR spectra of compounds **4**–**23** are available in the supplementary information.

### 3.2. In Vivo Anti-Tumor Effect of Dictyoceratin-C (**1**) and -A (**2**)

All procedures of animal experiment were approved as No. DOUYAKU24-8 by the Committee on Animal Experimentation of Osaka University. Murine sarcoma S180 cells (1 × 10^6^ cells/body), suspended in the RPMI1640 without FBS and kanamycin, were subcutaneously implanted into the right ventral flank of female ddY mice of 6 weeks old. After one week from implantation, dictyoceratin-C (**1**) or -A (**2**) was orally administered on every other day for two week (total seven times) as a suspension in 1% sodium carboxymethyl cellulose (CMC-Na). Then, tumor was isolated and weighed to calculate the ratio of inhibition. The control group and the groups treated with **1** or **2** consisted of six mice each in this study.

### 3.3. Design and Synthesis of Structure-Modified Analogues

#### 3.3.1. 4-Hydroxy-3-(((1*S*,2*R*,4a*R*,8a*R*)-1,2,4a-trimethyl-5-methylenedecahydronaphthalen-1-yl)methyl)benzoic acid (**4**)

Under an Ar atmosphere, KHMDS (5.9 mL, of a 0.5 M solution in toluene, 3.0 mmol) was added to a solution of Ph_3_PCH_3_Br (1.05 g, 3.0 mmol) in anhydrous THF (1.9 mL), and the whole mixture was stirred for 1 h at rt. The above solution (7.2 mL, about 2.7 mmol) was added dropwise to a solution of **3** (93.5 mg, 0.27 mmol) in anhydrous THF (0.9 mL) at 0 °C, and the whole mixture was stirred for 20 h at rt. Sat. NH_4_Cl aq. was added to the mixture, and the whole mixture was extracted with AcOEt. Removal of the solvent from the AcOEt extract under reduced pressure gave a crude product, which was purified by SiO_2_ column (*n*-hexane/AcOEt = 3:2) to give **4** (87.3 mg, 94%) as a white solid.

${\left[\alpha \right]}_{\text{D}}^{26}$ +33 (*c* = 1.22, MeOH). ^1^H-NMR (500 MHz, CDCl_3_) δ: 7.85–7.83 (2H, m), 6.76 (1H, d, *J* = 8.6 Hz), 4.42 (1H, s), 4.37 (1H, s), 2.70 (1H, d, *J* = 14.4 Hz), 2.63 (1H, d, *J* = 14.4 Hz), 2.34 (1H, td, *J* = 13.6, 5.1 Hz), 2.12–2.06 (2H, m), 1.95–1.91 (1H, m), 1.64–1.56 (1H, m), 1.48 (1H, dt, *J* = 12.4, 3.1 Hz), 1.44–1.19 (5H, m), 1.07 (3H, s), 1.04 (3H, d, *J* = 6.7 Hz), 0.95 (1H, dd, *J* = 12.2, 1.8 Hz), 0.89 (3H, s). ^13^C-NMR (125 MHz, CDCl_3_) δ: 171.9, 159.9, 159.4, 135.7, 130.1, 125.2, 121.2, 115.5, 102.9, 48.1, 42.1, 40.2, 37.1, 36.5, 36.3, 32.9, 27.9, 27.7, 23.3, 20.5, 17.62, 17.57. MS (ESI-TOF) *m*/*z*: 365 [M + Na]^+^. HRMS (ESI-TOF) *m*/*z*: 365.2093 calcd for C_22_H_30_O_3_Na; Found: 365.2083.

#### 3.3.2. Octyl 4-hydroxy-3-(((1*S*,2*R*,4a*R*,8a*R*)-1,2,4a-trimethyl-5-methylenedecahydronaphthalen-1-yl)methyl)benzoate (**5**)

The components SOCl_2_ (6.0 µL, 0.083 mmol) and DMF (1 drop) were added to a solution of **4** (4.8 mg, 0.014 mmol) in anhydrous THF (0.2 mL), and the whole mixture was stirred for 1 h at rt. *n*-Octanol (6.0 µL, 0.083 mmol) was added to the mixture, and the whole mixture was stirred for 7 h at 50 °C. Sat. NH_4_Cl aq. was added to the mixture, and the whole mixture was extracted with AcOEt. Removal of the solvent from the AcOEt extract under reduced pressure gave a crude product, which was purified by SiO_2_ column (CHCl_3_/MeOH/H_2_O = 200:3:1, lower phase) to give **5** (0.8 mg, 13%) as a white solid.

${\left[\alpha \right]}_{\text{D}}^{26}$ +14 (*c* = 0.31, CHCl_3_). ^1^H-NMR (500 MHz, CDCl_3_) δ: 7.78 (1H, dd, *J* = 8.2, 1.9 Hz), 7.75 (1H, d, *J* = 1.9 Hz), 6.73 (1H, d, *J* = 8.2 Hz), 5.17 (1H, s), 4.41 (1H, s), 4.37 (1H, s), 4.31–4.20 (2H, m), 2.67 (1H, d, *J* = 14.3 Hz), 2.62 (1H, d, *J* = 14.3 Hz), 2.34 (1H, td, *J* = 13.7, 5.2 Hz), 2.08 (2H, t, *J* = 11.7 Hz), 1.94–1.91 (1H, m), 1.76–1.70 (2H, m), 1.60 (2H, qd, *J* = 13.2, 6.6 Hz), 1.47–1.41 (5H, m), 1.31–1.23 (10H, m), 1.07 (3H, s), 1.03 (3H, d, *J* = 6.9 Hz), 0.96 (1H, dd, *J* = 12.0, 1.7 Hz), 0.88 (3H, t, *J* = 6.9 Hz), 0.88 (3H, s). ^13^C-NMR (125 MHz, CDCl_3_) δ: 166.6, 159.9, 158.5, 134.8, 129.3, 124.8, 122.5, 115.3, 102.9, 64.8, 47.9, 42.0, 40.2, 37.0, 36.5, 36.2, 33.0, 31.8, 29.3, 29.2, 28.8, 27.9, 27.7, 26.1, 23.3, 22.7, 20.5, 17.63, 17.60, 14.1. IR (KBr) : 2926, 1672, 1599, 1279 cm^−1^. MS (ESI-TOF) *m*/*z*: 477 [M + Na]^+^. HRMS (ESI-TOF) *m*/*z*: 477.3345 calcd for C_30_H_46_O_3_Na; Found: 477.3348.

#### 3.3.3. 4-Hydroxy-*N*-(prop-2-yn-1-yl)-3-(((1*S*,2*R*,4a*R*,8a*R*)-1,2,4a-trimethyl-5-methylenedecahydronaphthalen-1-yl)methyl)benzamide (**6**)

Propargylamine (7.0 µL, 0.10 mmol), EDCI·HCl (18.0 mg, 0.094 mmol) and HOBt (14.2 mg, 0.11 mmol) were added to a solution of **4** (10.8 mg, 0.032 mmol) in anhydrous DMF (0.3 mL), and the whole mixture was stirred for 6 h at rt. Sat. NH_4_Cl aq. was added to the mixture, and the whole mixture was extracted with AcOEt. Removal of the solvent from the AcOEt extract under reduced pressure gave a crude product, which was purified by SiO_2_ column (CHCl_3_/MeOH/H_2_O = 30:3:1, lower phase) to give **6** (7.2 mg, 60%) as a white solid.

${\left[\alpha \right]}_{\text{D}}^{26}$ +22.0 (*c* = 0.58, MeOH). ^1^H-NMR (500 MHz, acetone-*d*_6_) δ: 8.89 (1H, d, *J* = 4.0 Hz), 7.79 (1H, brs), 7.71 (1H, d, *J* = 2.3 Hz), 7.61 (1H, dd, *J* = 8.6, 2.3 Hz), 6.86 (1H, d, *J* = 8.6 Hz), 4.37 (1H, s), 4.33 (1H, s), 4.20–4.09 (2H, m), 2.72 (1H, d, *J* = 14.3 Hz), 2.68 (1H, d, *J* = 14.3 Hz), 2.63 (1H, t, *J* = 2.6 Hz), 2.35 (1H, td, *J* = 13.7, 5.2 Hz), 2.22 (1H, d, *J* = 13.2 Hz), 2.08–2.04 (1H, m), 1.90–1.85 (1H, m), 1.62–1.54 (1H, m), 1.51–1.36 (4H, m), 1.21–1.15 (1H, m), 1.07 (3H, s), 1.05 (3H, d, *J* = 5.7 Hz), 1.01 (1H, dd, *J* = 11.7, 2.0 Hz), 0.89 (3H, s). ^13^C-NMR (125 MHz, acetone-*d*_6_) δ: 166.8, 160.7, 160.0, 133.6, 127.0, 126.0, 125.8, 115.4, 103.2, 81.8, 71.6, 48.9, 42.7, 40.9, 37.6, 37.5, 37.1, 33.7, 29.2, 28.8, 28.4, 23.7, 20.9, 18.1, 18.0. IR (KBr): 3299, 2921, 1630, 1588, 1490, 1268 cm^−1^. MS (ESI-TOF) *m*/*z*: 402 [M + Na]^+^. HRMS (ESI-TOF) *m*/*z*: 402.2409 calcd for C_25_H_33_NO_2_Na; Found: 402.2418.

#### 3.3.4. Methyl 4-methoxy-3-(((1*S*,2*R*,4a*R*,8a*R*)-1,2,4a-trimethyl-5-methylenedecahydronaphthalen-1-yl)methyl)benzoate (**7**)

K_2_CO_3_ (7.5 mg, 0.054 mmol) and MeI (6.0 µL, 0.096 mmol) were added to a solution of **1** (6.8 mg, 0.018 mmol) in anhydrous DMF (0.2 mL), and the whole mixture was stirred for 12.5 h at rt. Sat. Na_2_S_2_O_3_ aq. was added to the mixture, and the whole mixture was extracted with AcOEt. Removal of the solvent from the AcOEt extract under reduced pressure gave a crude product, which was purified by SiO_2_ column (*n*-hexane/AcOEt = 8:1) to give **7** (7.6 mg, quantitative yield) as a white solid.

${\left[\alpha \right]}_{\text{D}}^{25}$ +23 (*c* = 0.68, CHCl_3_). ^1^H-NMR (500 MHz, CDCl_3_) δ: 7.87 (1H, dd, *J* = 8.9, 1.7 Hz), 7.74 (1H, d, *J* = 1.7 Hz), 6.82 (1H, d, *J* = 8.9 Hz), 4.40 (1H, s), 4.35 (1H, s), 3.87 (3H, s), 3.81 (3H, s), 2.68 (1H, d, *J* = 14.3 Hz), 2.65 (1H, d, *J* = 14.3 Hz), 2.33 (1H, td, *J* = 13.5, 5.2 Hz), 2.11–2.08 (2H, m), 1.95–1.92 (1H, m), 1.58–1.53 (2H, m), 1.45 (1H, dt, *J* = 12.4, 3.3 Hz), 1.41–1.30 (3H, m), 1.25–1.15 (1H, m), 1.06 (3H, s), 1.01 (3H, d, *J* = 6.3 Hz), 0.88 (1H, d, *J* = 12.6 Hz), 0.86 (3H, s). ^13^C-NMR (125 MHz, CDCl_3_) δ: 167.2, 162.1, 160.1, 134.1, 129.4, 127.4, 121.5, 109.6, 102.7, 55.1, 51.8, 48.0, 42.1, 40.2, 36.7, 36.6, 36.3, 33.1, 27.9, 27.7, 23.2, 20.6, 17.6, 17.5. IR (KBr): 2927, 1719, 1437, 1298, 1269 cm^−1^. MS (ESI-TOF) *m*/*z*: 393 [M + Na]^+^. HRMS (ESI-TOF) *m*/*z*: 393.2406 calcd for C_24_H_34_O_3_Na; Found: 393.2414.

#### 3.3.5. Methyl 3-hydroxy-4-(prop-2-yn-1-yloxy)-5-(((1*S*,2*R*,4a*R*,8a*R*)-1,2,4a-trimethyl-5-methylenedecahydronaphthalen-1-yl)methyl)benzoate (**8**); Methyl 4-hydroxy-3-(prop-2-yn-1-yloxy)-5-(((1*S*,2*R*,4a*R*,8a*R*)-1,2,4a-trimethyl-5-methylenedecahydronaphthalen-1-yl)methyl)benzoate (**9**)

K_2_CO_3_ (13.3 mg, 0.096 mmol) and propargyl bromide (9.0 µL, of a 80 wt% in toluene, 0.10 mmol) were added to a solution of **2** (30.6 mg, 0.082 mmol) in anhydrous DMF (1.65 mL), and the whole mixture was stirred for 3 h at 0 °C and for 13 h at rt. Sat. NH_4_Cl aq. was added to the mixture, and the whole mixture was extracted with AcOEt. Removal of the solvent from the AcOEt extract under reduced pressure gave a crude product, which was purified by SiO_2_ column (*n*-hexane/AcOEt = 5:1) to give **8** (11.9 mg, 35%) as a colorless amorphous and **9** (4.3 mg, 13%) as a colorless amorphous.

**8**: ${\left[\alpha \right]}_{\text{D}}^{26}$ +22 (*c* = 0.94, CHCl_3_). ^1^H-NMR (500 MHz, CDCl_3_) δ: 7.48 (1H, d, *J* = 2.0 Hz), 7.38 (1H, d, *J* = 2.0 Hz), 5.74 (1H, s), 4.59 (1H, dd, *J* = 15.5, 2.5 Hz), 4.53 (1H, dd, *J* = 15.5, 2.5 Hz), 4.40 (1H, s), 4.35 (1H, s), 3.87 (3H, s), 2.66 (1H, d, *J* = 13.7 Hz), 2.62 (1H, d, *J* = 13.7 Hz), 2.58 (1H, t, *J* = 2.5 Hz), 2.37–2.28 (1H, m), 2.10–2.07 (1H, m), 1.99 (1H, d, *J* = 13.2 Hz), 1.94–1.88 (1H, m), 1.57 (1H, qd, *J* = 13.0, 3.3 Hz), 1.45 (1H, dt, *J* = 12.4, 3.3 Hz), 1.42–1.33 (2H, m), 1.27–1.13 (3H, m), 1.05 (3H, s), 1.00 (3H, d, *J* = 6.3 Hz), 0.87 (3H, s), 0.84 (1H, dd, *J* = 12.0, 1.7 Hz). ^13^C-NMR (150 MHz, CDCl_3_) δ: 166.7, 159.8, 149.0, 148.8, 132.8, 126.2, 125.9, 115.2, 102.9, 78.2, 76.8, 61.0, 52.1, 47.8, 42.4, 40.1, 37.7, 36.4, 36.1, 32.9, 27.7, 27.5, 23.2, 20.6, 17.8, 17.6. IR (KBr): 3302, 2928, 1718, 1591, 1437, 1262 cm^−1^. MS (ESI-TOF) *m*/*z*: 433 [M + Na]^+^. HRMS (ESI-TOF) *m*/*z*: 433.2355 calcd for C_26_H_34_O_4_Na; Found: 433.2372.

**9**: ${\left[\alpha \right]}_{\text{D}}^{26}$ +14 (*c* = 0.36, CHCl_3_). ^1^H-NMR (500 MHz, CDCl_3_) δ: 7.493 (1H, d, *J* = 2.0 Hz), 7.486 (1H, d, *J* = 2.0 Hz), 6.12 (1H, s), 4.78 (2H, d, *J* = 2.2 Hz), 4.41 (1H, s), 4.36 (1H, s), 3.87 (3H, s), 2.68 (2H, s), 2.58 (1H, t, *J* = 2.3 Hz), 2.34 (1H, td, *J* = 13.7, 5.2 Hz), 2.14–2.07 (2H, m), 1.92 (1H, m), 1.66–1.52 (1H, m), 1.48–1.36 (3H, m), 1.32–1.17 (3H, m), 1.06 (3H, s), 1.03 (3H, d, *J* = 6.9 Hz), 0.94 (1H, dd, *J* = 11.7, 2.0 Hz), 0.87 (3H, s). ^13^C-NMR (125 MHz, CDCl_3_) δ: 167.0, 160.2, 149.7, 143.9, 128.5, 125.0, 120.4, 111.1, 102.6, 77.6, 76.5, 57.3, 51.9, 48.0, 42.1, 40.2, 36.9, 36.6, 36.3, 33.0, 27.9, 27.7, 23.1, 20.6, 17.7, 17.6. IR (KBr): 3295, 2926, 1713, 1437, 1300 cm^−1^. MS (ESI-TOF) *m*/*z*: 433 [M + Na]^+^. HRMS (ESI-TOF) *m*/*z*: 433.2355 calcd for C_26_H_34_O_4_Na; Found: 433.2364.

#### 3.3.6. Methyl 3-hydroxy-4-(nonyloxy)-5-(((1*S*,2*R*,4a*R*,8a*R*)-1,2,4a-trimethyl-5-methylenedecahydronaphthalen-1-yl)methyl)benzoate (**10**); Methyl 4-hydroxy-3-(nonyloxy)-5-(((1*S*,2*R*,4a*R*,8a*R*)-1,2,4a-trimethyl-5-methylenedecahydronaphthalen-1-yl)methyl)benzoate (**11**)

K_2_CO_3_ (9.2 mg, 0.067 mmol) and *n*-nonyl bromide (8.0 µL, 0.042 mmol) were added to a solution of **2** (12.6 mg, 0.034 mmol) in anhydrous DMF (0.34 mL), and the whole mixture was stirred for 3 h at rt. Sat. NH_4_Cl aq. was added to the mixture, and the whole mixture was extracted with AcOEt. Removal of the solvent from the AcOEt extract under reduced pressure gave a crude product, which was purified by SiO_2_ column (*n*-hexane/AcOEt = 30:1 to 5:1) to give **10** (3.6 mg, 21%) as a colorless amorphous and **11** (2.3 mg, 14%) as a colorless amorphous.

**10**: ${\left[\alpha \right]}_{\text{D}}^{26}$ +15 (*c* = 0.20, CHCl_3_). ^1^H-NMR (500 MHz, CDCl_3_) δ: 7.43 (1H, d, *J* = 1.7 Hz), 7.36 (1H, d, *J* = 1.7 Hz), 6.16 (1H, s), 4.41 (1H, s), 4.36 (1H, s), 4.07 (2H, t, *J* = 6.6 Hz), 3.86 (3H, s), 2.69 (1H, d, *J* = 14.0 Hz), 2.66 (1H, d, *J* = 14.0 Hz), 2.34 (1H, td, *J* = 13.5, 5.2 Hz), 2.13–2.07 (2H, m), 1.94–1.90 (1H, m), 1.85–1.79 (2H, m), 1.62–1.52 (3H, m), 1.48–1.19 (16H, m), 1.06 (3H, s), 1.03 (3H, d, *J* = 6.3 Hz), 0.96 (1H, dd, *J* = 12.0, 1.7 Hz), 0.89 (3H, t, *J* = 6.9 Hz), 0.87 (3H, s). ^13^C-NMR (150 MHz, CDCl_3_) δ: 167.2, 160.3, 149.4, 145.3, 127.4, 124.2, 120.3, 109.9, 102.6, 69.2, 51.9, 48.0, 42.1, 40.2, 36.9, 36.6, 36.4, 33.1, 31.9, 29.5, 29.4, 29.2, 29.1, 27.9, 27.7, 26.0, 23.1, 22.7, 20.7, 17.7, 17.6, 14.1. IR (KBr): 2925, 1716, 1437, 1301 cm^−1^. MS (ESI-TOF) *m*/*z*: 521 [M + Na]^+^. HRMS (ESI-TOF) *m*/*z*: 521.3607 calcd for C_32_H_50_O_4_Na; Found: 521.3616.

**11**: ${\left[\alpha \right]}_{\text{D}}^{26}$ +20 (*c* = 0.61, CHCl_3_). ^1^H-NMR (500 MHz, CDCl_3_) δ: 7.46 (1H, d, *J* = 2.0 Hz), 7.36 (1H, d, *J* = 2.0 Hz), 5.57 (1H, s), 4.40 (1H, s), 4.35 (1H, s), 3.87 (3H, s), 3.84–3.74 (2H, m), 2.66 (1H, d, *J* = 14.0 Hz), 2.58 (1H, d, *J* = 14.0 Hz), 2.31 (1H, td, *J* = 13.6, 5.5 Hz), 2.08 (1H, dd, *J* = 14.0, 3.2 Hz), 2.01 (1H, d, *J* = 12.6 Hz), 1.91–1.87 (1H, m), 1.83–1.77 (2H, m), 1.69–1.51 (3H, m), 1.47–1.11 (16H, m), 1.05 (3H, s), 0.99 (3H, d, *J* = 6.9 Hz), 0.89 (3H, t, *J* = 7.2 Hz), 0.87 (3H, s), 0.84 (1H, dd, *J* = 12.0, 1.7 Hz). ^13^C-NMR (150 MHz, CDCl_3_) δ: 166.8, 159.8, 149.9, 148.9, 132.2, 125.8, 125.5, 114.4, 102.9, 74.6, 52.0, 47.9, 42.3, 40.1, 37.3, 36.5, 36.1, 33.0, 31.8, 30.2, 29.5, 29.4, 29.2, 27.9, 27.6, 26.0, 23.1, 22.7, 20.6, 17.8, 17.5, 14.1. IR (KBr): 3394, 2926, 1722, 1437, 1335 cm^−1^. MS (ESI-TOF) *m*/*z*: 521 [M + Na]^+^. HRMS (ESI-TOF) *m*/*z*: 521.3607 calcd for C_32_H_50_O_4_Na; Found: 521.3630.

#### 3.3.7. Methyl 4-hydroxy-3-(((1*S*,2*R*,4a*S*,5*S*,8a*S*)-1,2,4a,5-tetramethyldecahydronaphthalen-1-yl)methyl)benzoate (**12a**); Methyl 4-hydroxy-3-(((1*S*,2*R*,4a*S*,5*R*,8a*S*)-1,2,4a,5-tetramethyldecahydronaphthalen-1-yl)methyl)benzoate (**12b**)

An amount of 10% Pd-C (6.8 mg) was added to a solution of **1** (15.4 mg, 0.041 mmol) in anhydrous MeOH (2.0 mL), and the whole mixture was stirred for 16 h under H_2_ atmosphere (baloon). The mixture was filtered through a short pad of Celite. Removal of the solvent from the filtrate under reduced pressure gave a crude product, which was purified by reversed-phase HPLC (Cosmosil AR-II, 85% aq. CH_3_CN, flow rate: 3 mL/min, UV: 220 nm) to give **12a** (7.5 mg, 51%) and **12b** (7.0 mg, 47%) as a white amorphous solid.

**12a**: ${\left[\alpha \right]}_{\text{D}}^{26}$ −3.8 (*c* = 0.66, CHCl_3_). ^1^H-NMR (500 MHz, CDCl_3_) δ: 7.83 (1H, d, *J* = 2.2 Hz), 7.79 (1H, dd, *J* = 8.5, 2.2 Hz), 6.78 (1H, d, *J* = 8.5 Hz), 5.22 (1H, s), 3.88 (3H, s), 2.63 (2H, s), 1.91–1.83 (2H, m), 1.69–1.55 (3H, m), 1.48–1.31 (3H, m), 1.28–1.21 (2H, m), 1.17–1.11 (2H, m), 1.04 (3H, s), 0.99 (3H, d, *J* = 6.3 Hz), 0.88 (1H, dt, *J* = 13.0, 3.2 Hz), 0.82 (3H, s), 0.70 (3H, d, *J* = 6.9 Hz). ^13^C-NMR (150 MHz, CDCl_3_) δ: 167.2, 158.8, 135.0, 129.2, 125.1, 121.8, 115.3, 51.8, 42.2, 41.1, 38.8, 36.8, 36.51, 36.48, 36.4, 29.1, 27.6, 23.5, 22.1, 21.2, 17.7, 17.5, 14.6. IR (KBr): 3291, 2934, 1680, 1601, 1420, 1285 cm^−1^. MS (ESI-TOF) *m*/*z*: 381 [M + Na]^+^. HRMS (ESI-TOF) *m*/*z*: 381.2406 calcd for C_23_H_34_O_3_Na; Found: 381.2413.

**12b**: ${\left[\alpha \right]}_{\text{D}}^{26}$ −6.5 (*c* = 0.67, CHCl_3_). ^1^H-NMR (500 MHz, CDCl_3_) δ: 7.80 (1H, d, *J* = 2.0 Hz), 7.78 (1H, dd, *J* = 8.4, 2.0 Hz), 6.76 (1H, d, *J* = 8.4 Hz), 5.25 (1H, s), 3.88 (3H, s), 2.63 (2H, s), 1.93 (1H, d, *J* = 12.0 Hz), 1.84–1.80 (1H, m), 1.67–1.42 (5H, m), 1.40–1.13 (5H, m), 1.01 (3H, d, *J* = 6.3 Hz), 0.90 (1H, dd, *J* = 12.3, 2.6 Hz), 0.84 (3H, s), 0.80 (3H, s), 0.65 (3H, d, *J* = 6.9 Hz). ^13^C-NMR (125 MHz, CDCl_3_) δ: 167.1, 158.7, 135.1, 129.2, 125.0, 122.0, 115.3, 51.8, 48.9, 45.4, 41.6, 38.3, 37.3, 36.9, 36.1, 30.8, 27.5, 26.4, 23.1, 17.73, 17.68, 15.0, 13.2. IR (KBr): 2928, 1717, 1692, 1283 cm^−1^. MS (ESI-TOF) *m*/*z*: 381 [M + Na]^+^. HRMS (ESI-TOF) *m*/*z*: 381.2406 calcd for C_23_H_34_O_3_Na; Found: 381.2413.

#### 3.3.8. Methyl 4-hydroxy-3-(((1*S*,2*R*,4a*R*,8a*R*)-1,2,4a,5-tetramethyl-1,2,3,4,4a,7,8,8a-octahydronaphthalen-1-yl)methyl)benzoate (**13**)

**Compound 1** (7.6 mg, 0.020 mmol) and RhCl_3_·3H_2_O (2.6 mg, 0.010 mmol) were mixed and dried. EtOH (1.0 mL) was added to the mixture, and the whole mixture was stirred for 12 h under reflux. Removal of the solvent from the mixture under reduced pressure gave a crude product, which was purified by SiO_2_ column (*n*-hexane/AcOEt = 5:1) and reversed-phase HPLC (Cosmosil AR-II, 85% aq. CH_3_CN, flow rate: 3 mL/min, UV: 220 nm) to give **13** (4.0 mg, 61%) as a white amorphous solid.

${\left[\alpha \right]}_{\text{D}}^{26}$ −27 (*c* = 0.39, CHCl_3_). ^1^H-NMR (500 MHz, CDCl_3_) δ: 7.80 (1H, d, *J* = 2.0 Hz), 7.77 (1H, dd, *J* = 8.6, 2.0 Hz), 6.74 (1H, d, *J* = 8.6 Hz), 5.27 (1H, s), 5.15 (1H, s), 3.87 (3H, s), 2.72 (1H, d, *J* = 14.6 Hz), 2.68 (1H, d, *J* = 14.6 Hz), 2.27–2.19 (1H, m), 2.13–2.08 (1H, m), 2.04 (1H, dd, *J* = 13.5, 6.6 Hz), 1.66–1.55 (2H, m), 1.50 (3H, d-like, *J* = 1.7 Hz), 1.38–1.25 (3H, m), 1.18 (1H, dd, *J* = 12.0, 1.1 Hz), 1.03 (3H, d, *J* = 6.3 Hz), 1.02 (3H, s), 0.93–0.88 (1H, m), 0.88 (3H, s). ^13^C-NMR (125 MHz, CDCl_3_) δ: 167.1, 158.7, 144.1, 135.2, 129.3, 125.0, 122.1, 120.6, 115.3, 51.8, 45.5, 41.7, 38.3, 37.2, 36.0, 35.7, 27.7, 26.0, 20.0, 19.9, 18.1, 17.7, 17.6. IR (KBr): 2944, 1688, 1603, 1433, 1285 cm^−1^. MS (ESI-TOF) *m*/*z*: 379 [M + Na]^+^. HRMS (ESI-TOF) *m*/*z*: 379.2249 calcd for C_23_H_32_O_3_Na; Found: 379.2252.

#### 3.3.9. *tert*-Butyl 3-(((4a′*R*,5′*R*,6′*R*,8a′*R*)-6′-hydroxy-5′,8a′-dimethyloctahydro-2′*H*-spiro[[1,3]dioxolane-2,1′-naphthalen]-5′-yl)methyl)-4-(methoxymethoxy)benzoate (**15**)

CeCl_3_·7H_2_O (545 mg, 1.46 mmol) was added to a solution of **14** (285 mg, 0.58 mmol) [9] in MeOH (5.8 mL), and the whole mixture was stirred for 5 min. NaBH_4_ (110 mg, 2.9 mmol) was added to the mixture at 0 °C, and the whole mixture was stirred for 2.5 h at 0 °C. Sat. NH_4_Cl aq. was added to the mixture, and the whole mixture was extracted with AcOEt. Removal of the solvent from the AcOEt extract under reduced pressure gave a crude product, which was purified by SiO_2_ column (*n*-hexane/AcOEt = 4:1) to give **15** (270 mg, 94%) as a white amorphous solid.

${\left[\alpha \right]}_{\text{D}}^{25}$ −8 (*c* = 1.07, CHCl_3_). ^1^H-NMR (500 MHz, CDCl_3_) δ: 7.87 (1H, dd, *J* = 8.9, 2.0 Hz), 7.68 (1H, d, *J* = 2.0 Hz), 7.14 (1H, d, *J* = 8.9 Hz), 5.28 (2H, s), 3.86 (1H, q, *J* = 6.1 Hz), 3.79–3.76 (2H, m), 3.71 (1H, q, *J* = 6.5 Hz), 3.52 (3H, s), 3.37 (1H, d, *J* = 3.4 Hz), 3.00 (1H, ddd, *J* = 10.3, 5.2, 3.4 Hz), 2.82 (1H, d, *J* = 14.3 Hz), 2.59 (1H, d, *J* = 14.3 Hz), 1.93 (1H, d, *J* = 13.2 Hz), 1.76–1.61 (2H, m), 1.58 (9H, s), 1.57–1.45 (5H, m), 1.31–1.22 (2H, m), 1.10 (3H, s), 0.97 (3H, s). ^13^C-NMR (125 MHz, CDCl_3_) δ: 165.6, 158.9, 133.6, 129.4, 126.7, 125.5, 113.2, 113.0, 95.1, 80.7, 73.0, 65.0, 64.7, 56.8, 43.7, 43.1, 41.4, 35.8, 30.4, 28.3, 28.2, 25.0, 22.6, 21.3, 17.2, 16.6. MS (ESI-TOF) *m*/*z*: 513 [M + Na]^+^. HRMS (ESI-TOF) *m*/*z*: 513.2828 calcd for C_28_H_42_O_7_Na; Found: 513.2829.

#### 3.3.10. 4-Hydroxy-3-(((1*R*,2*R*,4a*R*,8a*R*)-2-hydroxy-1,4a-dimethyl-5-oxodecahydronaphthalen-1-yl)methyl)benzoic acid (**16**)

An amount of 80% TFA (3.6 mL) was added to a solution of **15** (51.5 mg, 0.11 mmol) in THF (1.2 mL) at 0 °C, and the whole mixture was stirred for 3.5 h at 50 °C. Removal of the solvent from the mixture under reduced pressure gave a crude product, which was purified by SiO_2_ column (CHCl_3_/MeOH/H_2_O = 15:3:1, lower phase) to give **16** (36.6 mg, 97%) as a white amorphous solid.

${\left[\alpha \right]}_{\text{D}}^{25}$ +14 (*c* = 1.02, MeOH). ^1^H-NMR (500 MHz, acetone-*d*_6_) δ: 7.77 (1H, dd, *J* = 8.6, 2.0 Hz), 7.69 (1H, d, *J* = 2.0 Hz), 6.88 (1H, d, *J* = 8.6 Hz), 3.06–3.03 (1H, m), 2.81 (1H, d, *J* = 14.3 Hz), 2.76 (1H, d, *J* = 14.3 Hz), 2.65 (1H, td, *J* = 14.0, 7.1 Hz), 2.27 (1H, dd, *J* = 14.0, 2.0 Hz), 2.18–2.13 (1H, m), 2.08–2.07 (1H, m), 1.96 (1H, qd, *J* = 13.0, 3.6 Hz), 1.76–1.59 (3H, m), 1.44 (1H, dt, *J* = 14.1, 3.3 Hz), 1.35–1.25 (2H, m), 1.20 (3H, s), 1.15 (1H, dd, *J* = 12.3, 2.0 Hz), 1.10 (3H, s). ^13^C-NMR (125 MHz, acetone-*d*_6_) δ: 213.7, 167.5, 162.0, 134.8, 130.7, 124.5, 122.6, 117.1, 73.9, 49.0, 47.2, 44.3, 37.7, 37.3, 32.0, 26.1, 26.0, 21.7, 19.6, 17.2. MS (ESI-TOF) *m*/*z*: 369 [M + Na]^+^. HRMS (ESI-TOF) *m*/*z*: 369.1678 calcd for C_20_H_26_O_5_Na; Found: 369.1680.

#### 3.3.11. Methyl 4-hydroxy-3-(((1*R*,2*R*,4a*R*,8a*R*)-2-hydroxy-1,4a-dimethyl-5-oxodecahydronaphthalen-1-yl)methyl)benzoate (**17**)

SOCl_2_ (0.02 mL, 0.28 mmol) was added to a solution of **16** (23.6 mg, 0.068 mmol) in anhydrous MeOH (0.7 mL), and the whole mixture was stirred for 6.5 h at 45 °C. Removal of the solvent from the mixture under reduced pressure gave a crude product, which was purified by SiO_2_ column (CHCl_3_/MeOH/H_2_O = 60:3:1, lower phase) to give **17** (23.9 mg, 98%) as a white solid.

${\left[\alpha \right]}_{\text{D}}^{25}$ +12 (*c* = 0.82, MeOH). ^1^H-NMR (500 MHz, acetone-*d*_6_) δ: 7.74 (1H, dd, *J* = 8.2, 1.9 Hz), 7.67 (1H, d, *J* = 1.9 Hz), 6.89 (1H, d, *J* = 8.2 Hz), 3.80 (3H, s), 3.04 (1H, dd, *J* = 10.3, 5.7 Hz), 2.88 (1H, br), 2.82 (1H, d, *J* = 14.9 Hz), 2.77 (1H, d, *J* = 14.9 Hz), 2.66 (1H, td, *J* = 14.0, 7.0 Hz), 2.27 (1H, dd, *J* = 14.0, 2.0 Hz), 2.22–2.17 (1H, m), 2.10–2.06 (1H, m), 2.03–1.97 (1H, m), 1.75–1.55 (3H, m), 1.44 (1H, dt, *J* = 14.3, 3.4 Hz), 1.33–1.23 (2H, m), 1.21 (3H, s), 1.16–1.13 (1H, dd, *J* = 12.3, 2.3 Hz), 1.11 (3H, s). ^13^C-NMR (125 MHz, acetone-*d*_6_) δ: 213.6, 167.0, 162.1, 134.5, 130.3, 124.6, 122.3, 117.1, 73.9, 51.9, 49.0, 47.2, 44.3, 37.7, 37.3, 32.0, 26.1, 26.0, 21.7, 19.6, 17.2. MS (ESI-TOF) *m*/*z*: 383 [M + Na]^+^. HRMS (ESI-TOF) *m*/*z*: 383.1834 calcd for C_21_H_28_O_5_Na; Found: 383.1847.

#### 3.3.12. Methyl 4-hydroxy-3-(((1*R*,2*R*,4a*R*,8a*S*)-2-hydroxy-1,4a-dimethyl-5-methylenedecahydronaphthalen-1-yl)methyl)benzoate (**18**)

Under an Ar atmosphere, KHMDS (0.9 mL, of a 0.5 M solution in toluene, 0.45 mmol) was added to a solution of Ph_3_PCH_3_Br (165 mg, 0.46 mmol) in anhydrous THF (0.22 mL), and the whole mixture was stirred for 1 h at rt. The above solution was added dropwise to a solution of **17** (10.9 mg, 0.030 mmol) in anhydrous THF (0.1 mL) at 0 °C, and the whole mixture was stirred for 1 h at rt. Sat. NH_4_Cl aq. was added to the mixture, and the whole mixture was extracted with AcOEt. Removal of the solvent from the AcOEt extract under reduced pressure gave a crude product, which was purified by SiO_2_ column (*n*-hexane/AcOEt = 3:1) to give **18** (5.6 mg, 39%) as a white solid.

${\left[\alpha \right]}_{\text{D}}^{25}$ −32 (*c* = 0.55, CHCl_3_). ^1^H-NMR (500 MHz, CDCl_3_) δ: 7.81 (1H, dd, *J* = 8.5, 1.9 Hz), 7.63 (1H, d, *J* = 1.9 Hz), 6.89 (1H, d, *J* = 8.5 Hz), 4.45 (1H, s), 4.37 (1H, s), 3.85 (3H, s), 3.12 (1H, dd, *J* = 11.7, 4.3 Hz), 2.71 (2H, s), 2.31 (1H, td, *J* = 13.7, 5.7 Hz), 2.16–2.09 (2H, m), 2.04–2.00 (1H, m), 1.78 (1H, qd, *J* = 12.6, 3.2 Hz), 1.71–1.56 (4H, m), 1.48–1.40 (1H, m), 1.36 (1H, td, *J* = 13.3, 3.8 Hz), 1.11 (3H, s), 1.03 (3H, s), 1.00 (1H, dd, *J* = 12.6, 2.3 Hz). ^13^C-NMR (150 MHz, CDCl_3_) δ: 167.1, 160.9, 158.5, 133.9, 129.8, 123.2, 121.7, 116.6, 103.2, 74.7, 51.8, 46.2, 43.1, 39.5, 36.6, 34.8, 32.7, 27.4, 26.9, 22.1, 21.2, 16.3. IR (KBr): 3210, 2938, 1721, 1435, 1289 cm^−1^. MS (ESI-TOF) *m*/*z*: 381 [M + Na]^+^. HRMS (ESI-TOF) *m*/*z*: 381.2042 calcd for C_22_H_30_O_4_Na; Found: 381.2032.

#### 3.3.13. *tert*-Butyl 3-(((4a′*R*,5′*R*,6′*R*,8a′*R*)-5′,8a′-dimethyl-6′-(((methylthio)carbonothioyl)oxy)octahydro-2′*H*-spiro[[1,3]dioxolane-2,1′-naphthalen]-5′-yl)methyl)-4-(methoxymethoxy)benzoate (**19**)

Under an Ar atmosphere, NaH (91.0 mg, 60% suspension in oil, 2.3 mmol) was added to a solution of **15** (136 mg, 0.28 mmol) in anhydrous THF (2.8 mL) at 0 °C, and the whole mixture was stirred for 30 min at rt. CS_2_ (0.17 mL, 2.8 mmol) was added dropwise to the mixture, and the whole mixture was stirred for 50 min at rt. MeI (0.21 mL, 3.4 mmol) was added to the mixture, and the whole mixture was stirred for 5 h at 50 °C. Sat. Na_2_S_2_O_3_ aq. was added to the mixture, and the whole mixture was extracted with AcOEt. Removal of the solvent from the AcOEt extract under reduced pressure gave a crude product, which was purified by SiO_2_ column (*n*-hexane/AcOEt = 5:1) to give **19** (152 mg, 94%) as a white amorphous solid.

${\left[\alpha \right]}_{\text{D}}^{25}$ −18 (*c* = 1.17, CHCl_3_). ^1^H-NMR (500 MHz, CDCl_3_) δ: 7.79 (1H, dd, *J* = 8.6, 2.3 Hz), 7.68 (1H, d, *J* = 2.3 Hz), 7.02 (1H, d, *J* = 8.6 Hz), 5.18 (2H, dd, *J* = 8.0, 6.9 Hz), 5.13 (1H, dd, *J* = 11.2, 4.3 Hz), 3.92–3.88 (3H, m), 3.79–3.77 (1H, m), 3.43 (3H, s), 2.96 (1H, d, *J* = 14.0 Hz), 2.64 (1H, d, *J* = 14.0 Hz), 2.28–2.24 (1H, m), 2.19 (3H, s), 1.88 (1H, d, *J* = 12.6 Hz), 1.80 (1H, dd, *J* = 12.0, 2.3 Hz), 1.77–1.61 (3H, m), 1.58 (9H, s), 1.54–1.44 (4H, m), 1.39–1.35 (1H, m), 1.17 (3H, s), 1.15 (3H, s). ^13^C-NMR (125 MHz, CDCl_3_) δ: 213.4, 165.8, 158.8, 133.6, 129.3, 127.1, 124.6, 113.0, 112.9, 93.9, 88.7, 80.4, 65.2, 64.9, 56.2, 45.7, 43.2, 38.9, 30.4, 28.2 (3C), 27.7, 22.8, 21.6, 21.0, 17.8, 17.0, 16.9. MS (ESI-TOF) *m*/*z*: 603 [M + Na]^+^. HRMS (ESI-TOF) *m*/*z*: 603.2426 calcd for C_30_H_44_O_7_S_2_Na; Found: 603.2449.

#### 3.3.14. *tert*-Butyl 3-(((4a′*R*,5′*R*,8a′*R*)-5′,8a′-dimethyloctahydro-2′*H*-spiro[[1,3]dioxolane-2,1′-naphthalen]-5′-yl)methyl)-4-(methoxymethoxy)benzoate (**20**)

Under an Ar atmosphere, *n*Bu_3_SnH (0.21 mL, 0.80 mmol) and AIBN (34.5 mg, 0.21 mmol) were added to a solution of **19** (138 mg, 0.24 mmol) in anhydrous toluene (3.4 mL), and the whole mixture was stirred for 3 h at 80 °C. H_2_O was added to the mixture, and the whole mixture was extracted with AcOEt. Removal of the solvent from the AcOEt extract under reduced pressure gave a crude product, which was purified by SiO_2_ column (*n*-hexane/AcOEt = 8:1) to give **20** (96.6 mg, 86%) as a white amorphous solid.

${\left[\alpha \right]}_{\text{D}}^{25}$ +21 (*c* = 1.26, CHCl_3_). ^1^H-NMR (500 MHz, CDCl_3_) δ: 7.79 (1H, dd, *J* = 8.6, 2.3 Hz), 7.70 (1H, d, *J* = 2.3 Hz), 7.07 (1H, d, *J* = 8.6 Hz), 5.21 (1H, d, *J* = 6.9 Hz), 5.18 (1H, d, *J* = 6.9 Hz), 3.94–3.89 (3H, m), 3.83–3.79 (1H, m), 3.46 (3H, s), 2.88 (1H, d, *J* = 13.0 Hz), 2.40 (1H, d, *J* = 13.0 Hz), 1.81 (1H, d, *J* = 13.2 Hz), 1.75–1.64 (2H, m), 1.61–1.58 (1H, m), 1.57 (9H, s), 1.52–1.50 (1H, m), 1.44–1.21 (8H, m), 1.10 (3H, s), 0.85 (3H, s). ^13^C-NMR (125 MHz, CDCl_3_) δ: 165.9, 159.5, 134.1, 128.9, 127.9, 124.3, 113.5, 112.7, 94.2, 80.4, 65.2, 64.8, 56.2, 47.6, 43.8, 42.3, 37.9, 37.6, 30.6, 30.2, 28.2 (3C), 23.0, 20.9, 19.3, 17.8, 17.1. MS (ESI-TOF) *m*/*z*: 497 [M + Na]^+^. HRMS (ESI-TOF) *m*/*z*: 497.2879 calcd for C_28_H_42_O_6_Na; Found: 497.2897.

#### 3.3.15. 3-(((1*R*,4a*R*,8a*R*)-1,4a-Dimethyl-5-oxodecahydronaphthalen-1-yl)methyl)-4-hydroxybenzoic acid (**21**)

An amount of 80% TFA (4.0 mL) was added to a solution of **20** (74.9 mg, 0.16 mmol) in THF (2.0 mL), and the whole mixture was stirred for 2.5 h at 50 °C. Removal of the solvent from the mixture under reduced pressure gave a crude product, which was purified by SiO_2_ column (CHCl_3_/MeOH/H_2_O = 30:3:1, lower phase) to give **21** (50.0 mg, 93%) as a white amorphous solid.

${\left[\alpha \right]}_{\text{D}}^{25}$ +46 (*c* = 1.13, MeOH). ^1^H-NMR (500 MHz, acetone-*d*_6_) δ: 7.75 (1H, dd, *J* = 8.6, 2.3 Hz), 7.74 (1H, d, *J* = 2.3 Hz), 6.92 (1H, d, *J* = 8.6 Hz), 2.77 (1H, d, *J* = 13.2 Hz), 2.70–2.60 (1H, m), 2.61 (1H, d, *J* = 13.2 Hz), 2.16–2.07 (2H, m), 1.83 (1H, qd, *J* = 12.9, 3.7 Hz), 1.69–1.40 (4H, m), 1.37–1.26 (5H, m), 1.20 (3H, s), 1.05 (3H, s). ^13^C-NMR (125 MHz, acetone-*d*_6_) δ: 214.3, 161.1, 135.6, 130.3, 125.8, 122.0, 115.7, 78.1, 51.1, 49.7, 42.5, 39.6, 37.88, 37.86, 33.8, 26.6, 21.8, 21.4, 19.4, 18.2. MS (ESI-TOF) *m*/*z*: 353 [M + Na]^+^. HRMS (ESI-TOF) *m*/*z*: 353.1729 calcd for C_20_H_26_O_4_Na; Found: 353.1730.

#### 3.3.16. Methyl 3-(((1*R*,4a*R*,8a*R*)-1,4a-dimethyl-5-oxodecahydronaphthalen-1-yl)methyl)-4-hydroxybenzoate (**22**)

SOCl_2_ (0.03 mL, 0.413 mmol) was added to a solution of **21** (30.3 mg, 0.092 mmol) in anhydrous MeOH (0.92 mL), and the whole mixture was stirred for 15 h at 50 °C. Removal of the solvent from the mixture under reduced pressure gave a crude product, which was purified by SiO_2_ column (*n*-hexane/AcOEt = 3:1) to give **22** (27.5 mg, 87%) as a white solid.

${\left[\alpha \right]}_{\text{D}}^{25}$ +46 (*c* = 0.73, MeOH). ^1^H-NMR (500 MHz, acetone-*d*_6_) δ: 9.10 (1H, br s), 7.72 (1H, dd, *J* = 8.6, 1.7 Hz), 7.70 (1H, d, *J* = 1.7 Hz), 6.91 (1H, d, *J* = 8.6 Hz), 3.81 (3H, s), 2.77 (1H, d, *J* = 13.2 Hz), 2.66 (1H, td, *J* = 13.7, 6.9 Hz), 2.60 (1H, d, *J* = 13.2 Hz), 2.16–2.08 (3H, m), 1.83 (1H, qd, *J* = 13.2, 3.7 Hz), 1.68–1.25 (7H, m), 1.20 (3H, s), 1.11 (1H, t, *J* = 7.2 Hz), 1.05 (3H, s). ^13^C-NMR (125 MHz, acetone-*d*_6_) δ: 214.2, 167.1, 161.1, 135.2, 130.0, 125.9, 121.8, 115.8, 51.8, 51.1, 49.7, 42.4, 39.6, 37.89, 37.86, 33.8, 26.6, 21.8, 21.4, 19.4, 18.2. MS (ESI-TOF) *m*/*z*: 367 [M + Na]^+^. HRMS (ESI-TOF) *m*/*z*: 367.1885 calcd for C_21_H_28_O_4_Na; Found: 367.1875.

#### 3.3.17. Methyl 3-(((1*R*,4a*R*,8a*R*)-1,4a-dimethyl-5-methylenedecahydronaphthalen-1-yl)methyl)-4-hydroxybenzoate (**23**)

Under an Ar atmosphere, KHMDS (0.57 mL, of a 0.5 M solution in toluene, 0.29 mmol) was added to Ph_3_PCH_3_Br (114 mg, 0.32 mmol), and the whole mixture was stirred for 1 h at rt. The above solution was added dropwise to a solution of **22** (14.5 mg, 0.042 mmol) in anhydrous THF (0.21 mL) at 0 °C, and the whole mixture was stirred for 3.5 h at rt. Sat. NH_4_Cl aq. was added to the mixture, and the whole mixture was extracted with AcOEt. Removal of the solvent from the AcOEt extract under reduced pressure gave a crude product, which was purified by SiO_2_ column (*n*-hexane/AcOEt = 4:1) to give **23** (13.7 mg, 95%) as a white solid.

${\left[\alpha \right]}_{\text{D}}^{26}$ +60 (*c* = 0.89, CHCl_3_). ^1^H-NMR (500 MHz, CDCl_3_) δ: 7.78 (1H, dd, *J* = 8.3, 2.3 Hz), 7.73 (1H, d, *J* = 2.3 Hz), 6.78 (1H, d, *J* = 8.3 Hz), 5.28 (1H, br s), 4.47 (1H, s), 4.46 (1H, s), 3.87 (3H, s), 2.68 (1H, d, *J* = 13.5 Hz), 2.44 (1H, d, *J* = 13.5 Hz), 2.36 (1H, td, *J* = 13.7, 5.2 Hz), 2.15–2.11 (1H, m), 1.97–1.92 (2H, m), 1.62–1.46 (4H, m), 1.44–1.23 (4H, m), 1.11 (3H, s), 1.09 (1H, d, *J* = 12.0 Hz), 0.95 (3H, s). ^13^C-NMR (125 MHz, CDCl_3_) δ: 167.0, 160.2, 158.4, 134.9, 129.3, 124.9, 122.1, 115.2, 102.5, 51.9, 42.5, 40.2, 38.7, 38.1, 37.0, 33.0, 29.7, 28.4, 22.3, 20.8, 20.3, 18.4. IR (KBr): 2928, 1688, 1603, 1429, 1283 cm^−1^. MS (ESI-TOF) *m*/*z*: 365 [M + Na]^+^. HRMS (ESI-TOF) *m*/*z*: 365.2093 calcd for C_22_H_30_O_3_Na; Found: 365.2089.

### 3.4. Biological Evaluation of Analogues

#### 3.4.1. Cell Cultures

Human prostate cancer cell line DU145 was cultured in RPMI 1640 supplemented with heat-inactivated 10% fetal bovine serum (FBS) and kanamycin (50 μg/mL) in a humidified atmosphere of 5% CO_2_ at 37 °C.

#### 3.4.2. Assay for Anti-Proliferative Activity under Hypoxic Condition

The DU145 cells in the culture medium were plated into each well of 96-well plates (1 × 10^4^ cells/well/200 μL) in a humidified atmosphere of 5% CO_2_ at 37 °C (normoxic condition). After 4 h, the plates were incubated for 12 h in the hypoxic condition (94% nitrogen, 5% CO_2_, and 1% O_2_) inducing hypoxia-related genes such as HIF-1α. Then, testing compounds were added, and the plates were incubated for additional 24 h in the hypoxic condition. The cell proliferation was detected by MTT method as previously described [8]. The growth inhibition rate was calculated as percentage of parallel negative controls. The anti-proliferative activities of the testing compounds under normoxic condition were also evaluated by the MTT method.

## 4. Conclusions

In summary, we demonstrated the potential of dictyoceratin-C (**1**) and A (**2**) as anticancer drug leads by evaluating their *in vivo* antitumor activity. In addition, we investigated the SAR of **1** and **2** by using our synthetic methodology and concluded that the characteristic moieties of **1** were important for hypoxia-selective growth inhibition. This evidence conflicts with a previous study, which showed that the unnatural enantiomers of **1** and **2** also exhibited similar hypoxia-selective growth inhibitory activity. In order to clarify this contradiction, we focused on determining the target molecules of **1** and **2**. Substitution of the methyl ester of **1** with the more physiologically stable propargyl amide was found to be effective. Moreover, the propargyl group could be a useful tag for further functionalization through click chemistry, leading to the synthesis of probe molecules for target identification of **1** and **2**. These studies are now underway.

## Supplementary Files

Supplementary File 1
