# Supplementary material for: Structure-Activity Relationship and in Vivo Anti-Tumor Evaluations of Dictyoceratin-A and -C, Hypoxia-Selective Growth Inhibitors from Marine Sponge
Source: Mar Drugs. 2015 Dec 16;13(12):7419–32. doi: 10.3390/md13127074 (PMC4699247; doi:10.3390/md13127074)
Supplement: Supplementary File 1 [file marinedrugs-13-07074-s001.pdf]

Supplementary Materials: Structure-Activity Relationship and *in Vivo* Anti-Tumor Evaluations of Dictyoceratin-A and -C, Hypoxia-Selective Growth Inhibitors from Marine Sponge

Yuji Sumii, Naoyuki Kotoku *, Akinori Fukuda, Takashi Kawachi, Masayoshi Arai and Motomasa Kobayashi *

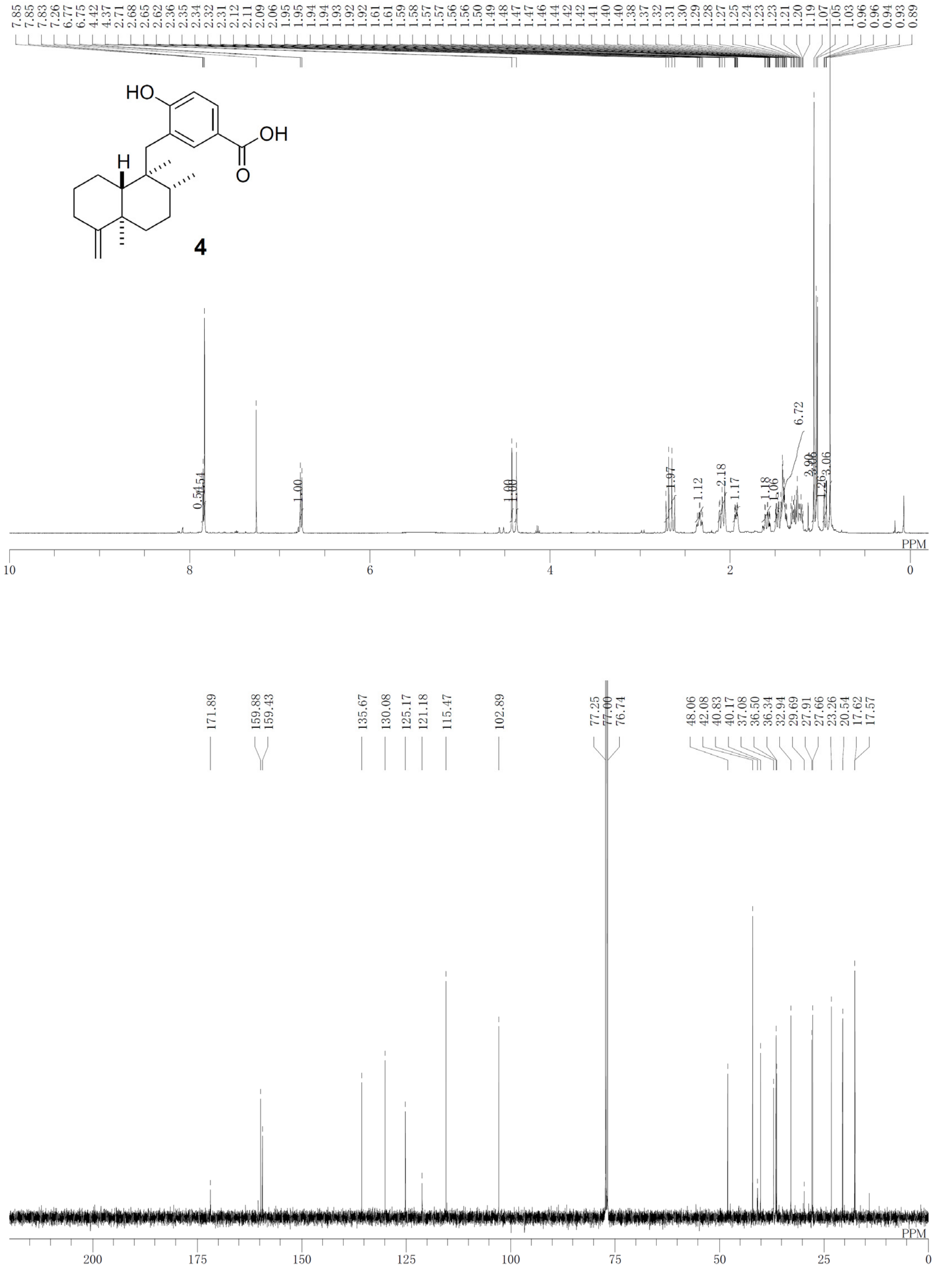

Figure S1. ^{1}H- and ^{13}C-NMR spectra of compound **4**.

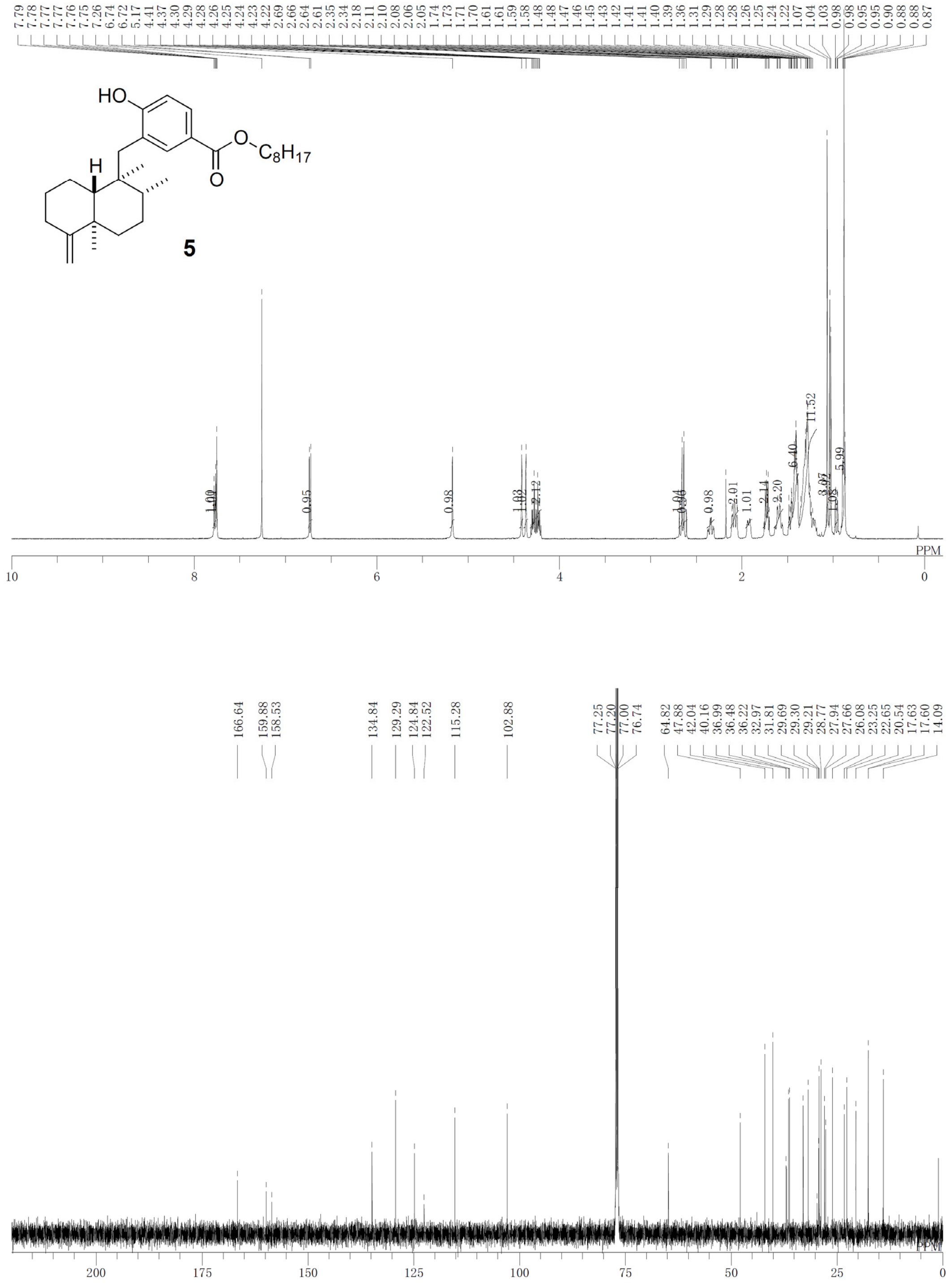

Figure S2. ^{1}H- and ^{13}C-NMR spectra of compound **5**.

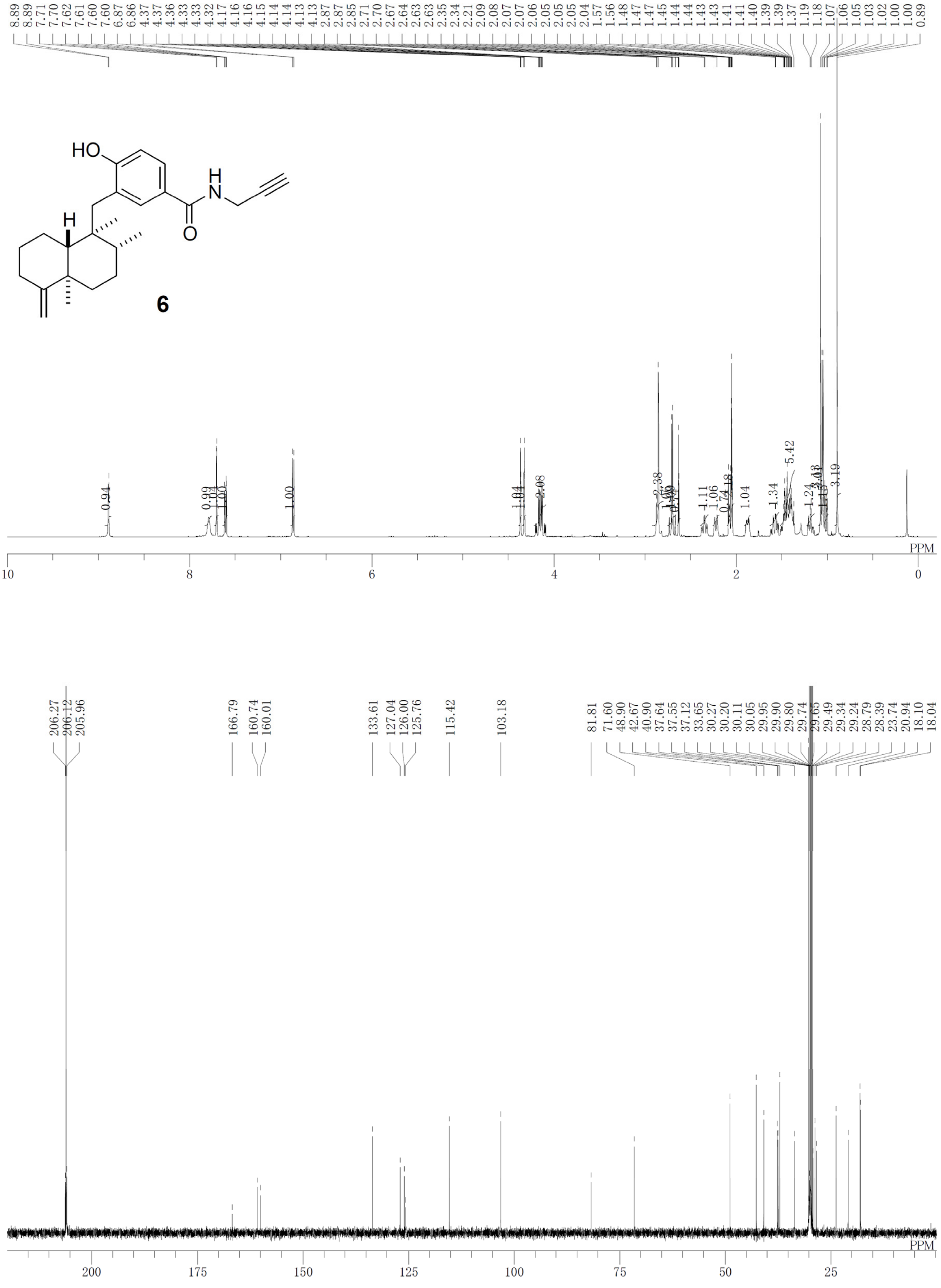

Figure S3. ^{1}H- and ^{13}C-NMR spectra of compound **6**.

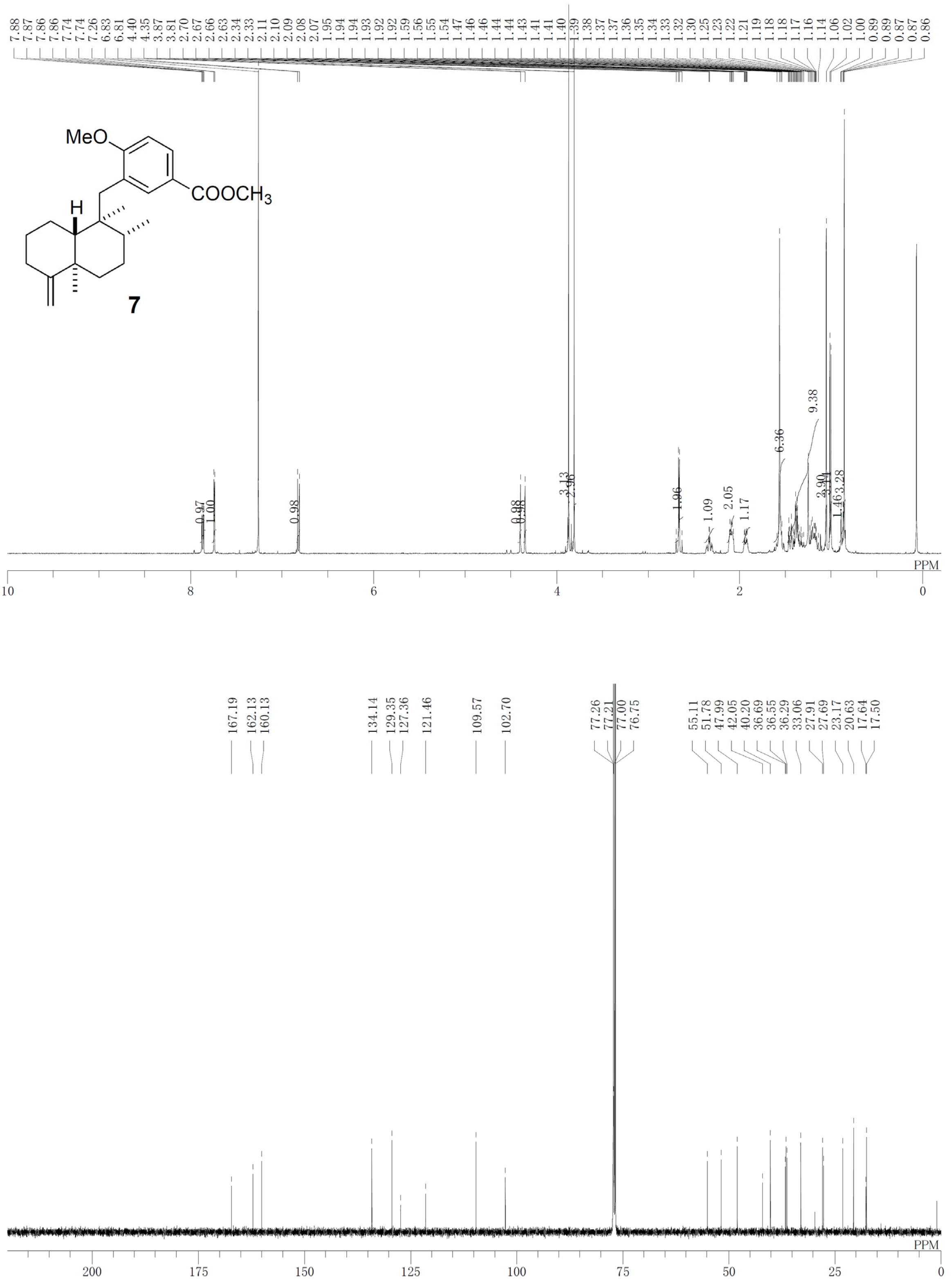

Figure S4. ^{1}H- and ^{13}C-NMR spectra of compound **7**.

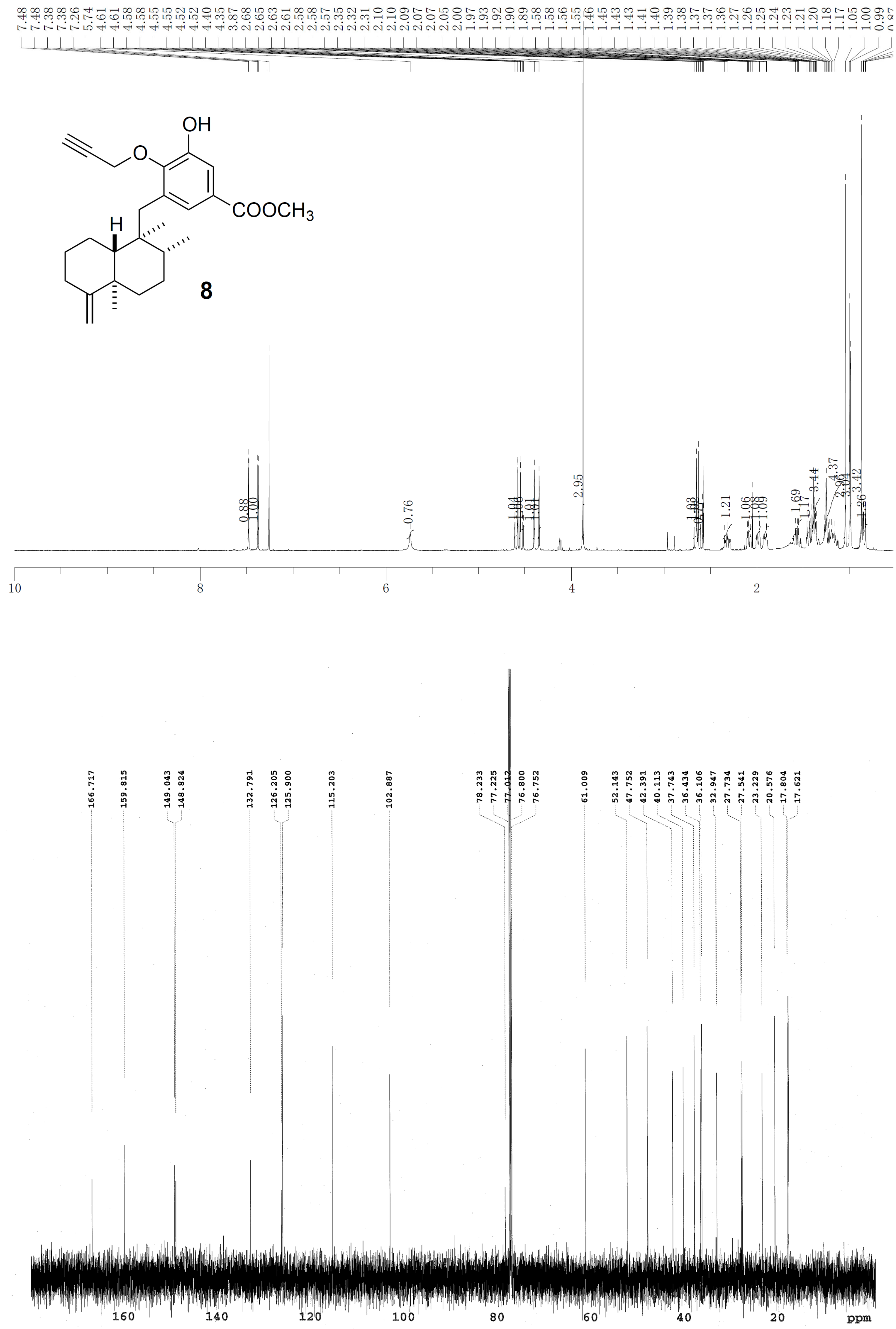

Figure S5. ^{1}H- and ^{13}C-NMR spectra of compound **8**.

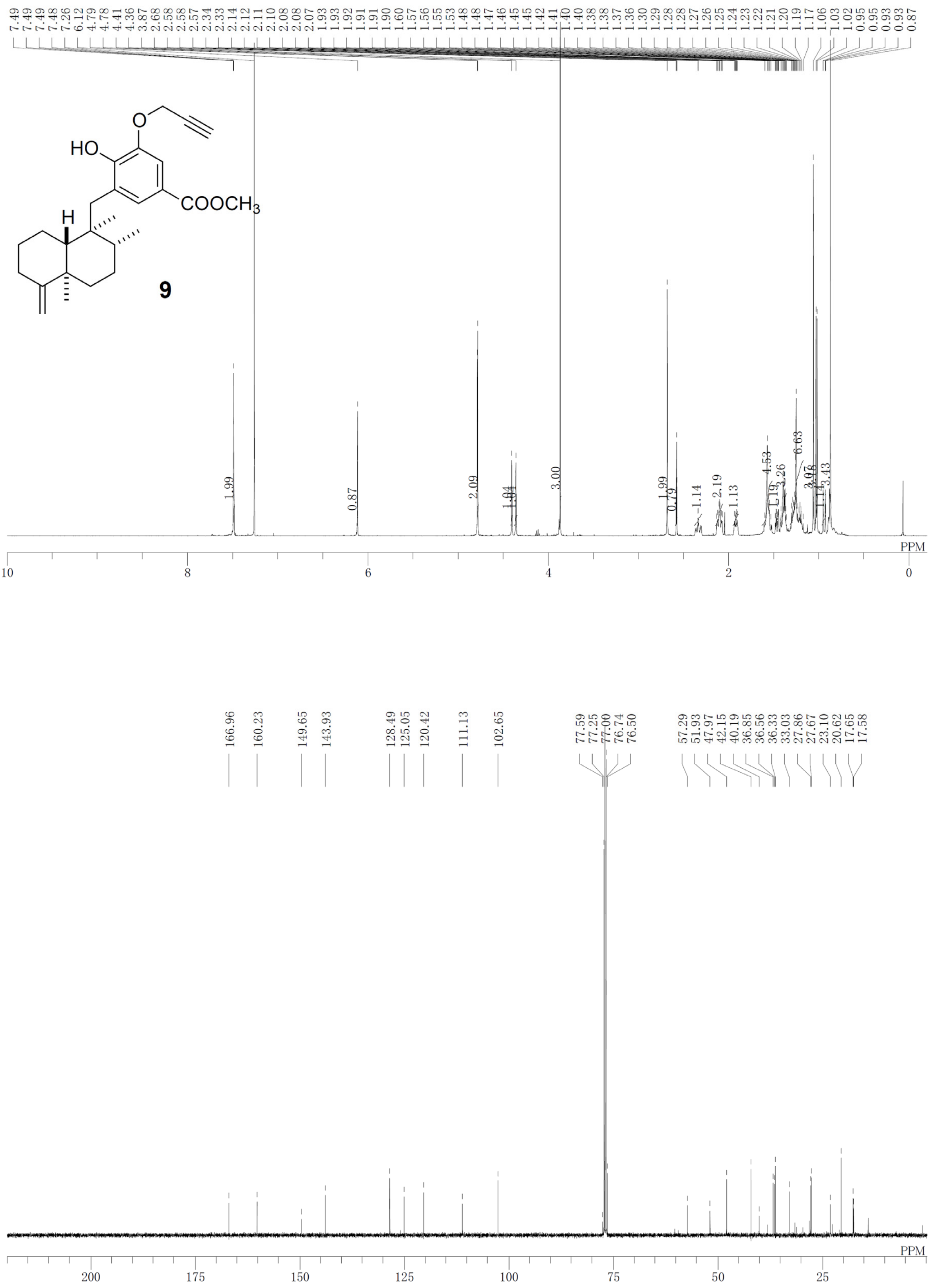

Figure S6. ^{1}H- and ^{13}C-NMR spectra of compound **9**.

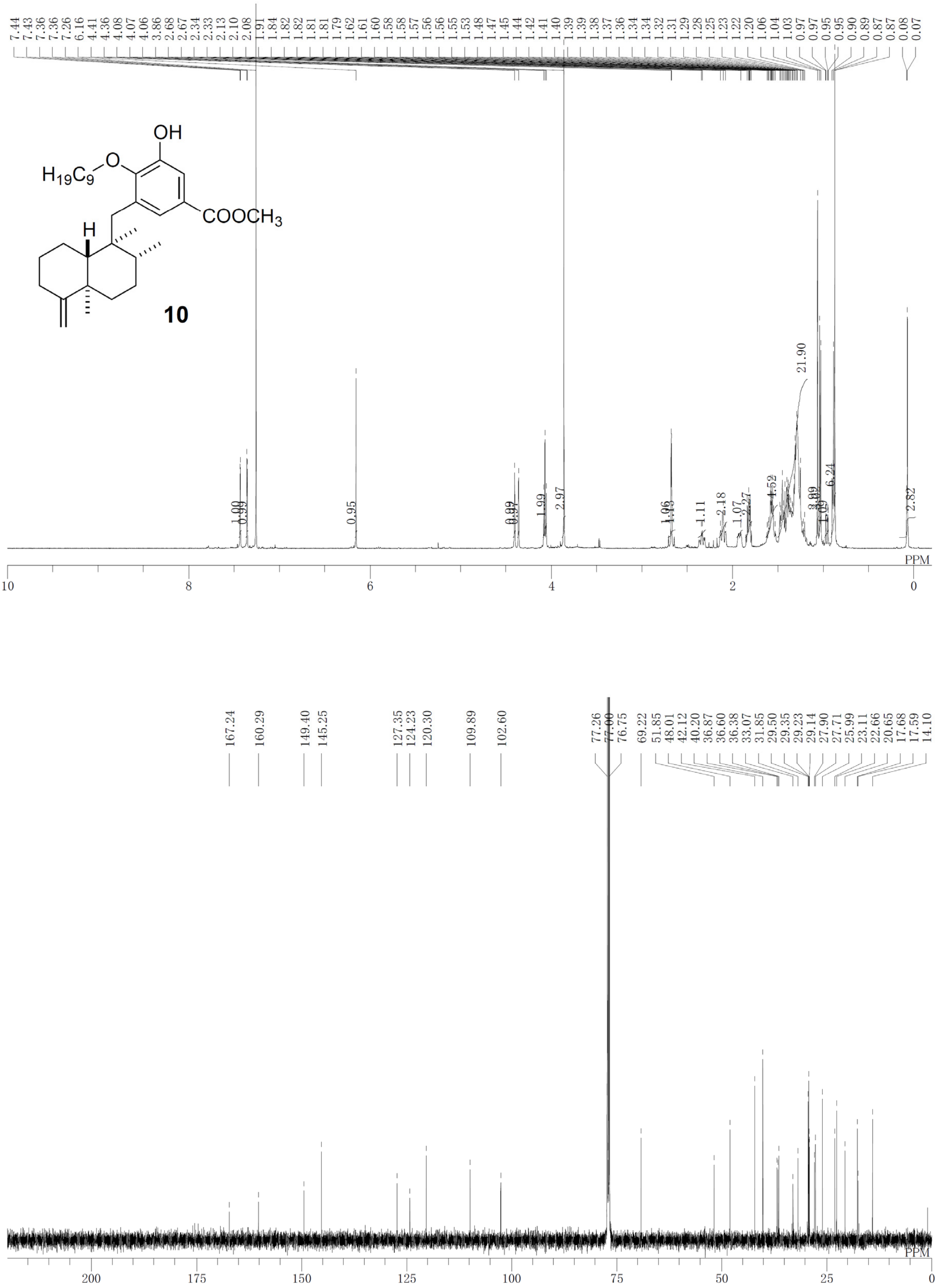

Figure S7. ^{1}H- and ^{13}C-NMR spectra of compound **10**.

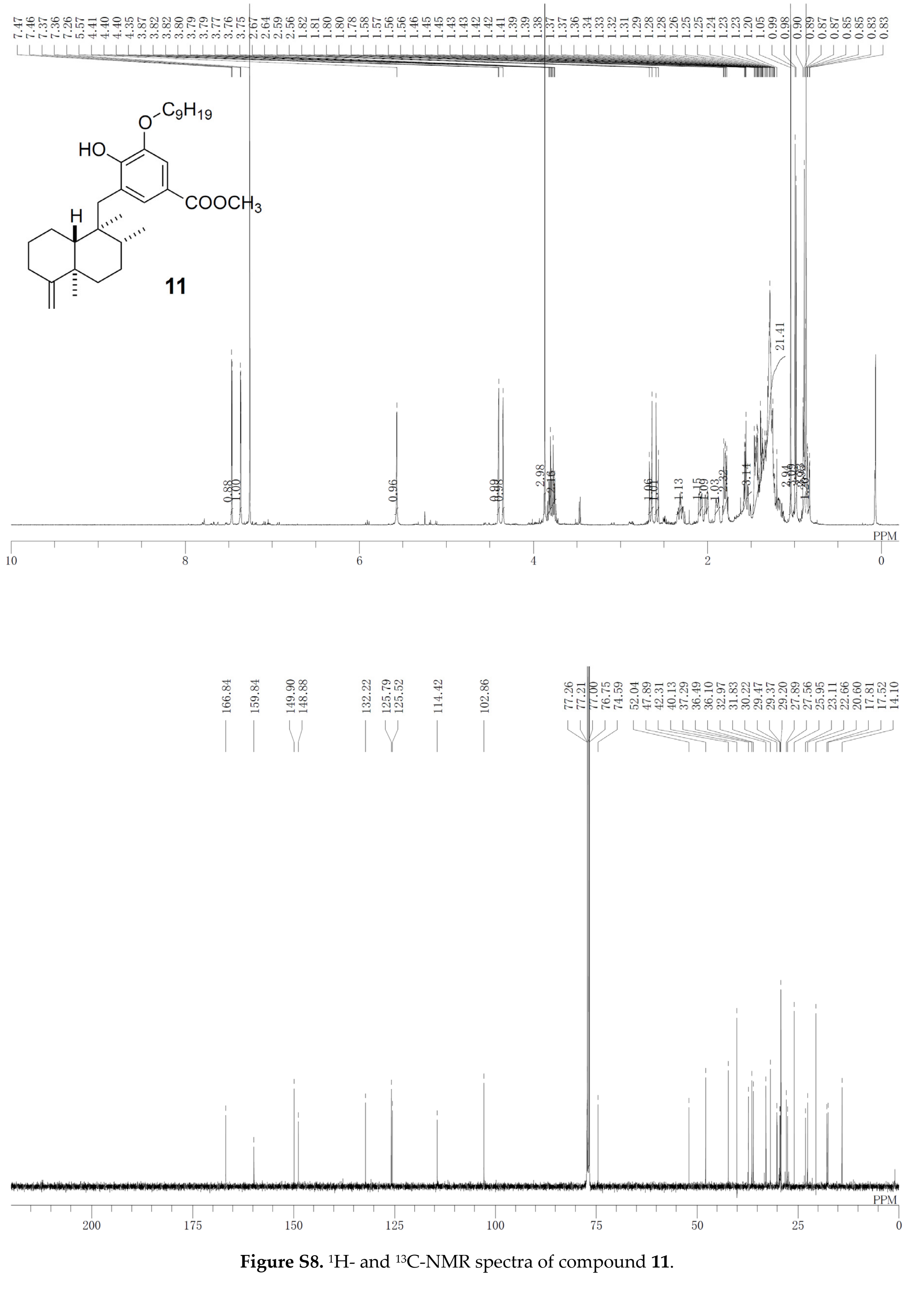

Figure S8. ^{1}H- and ^{13}C-NMR spectra of compound **11**.

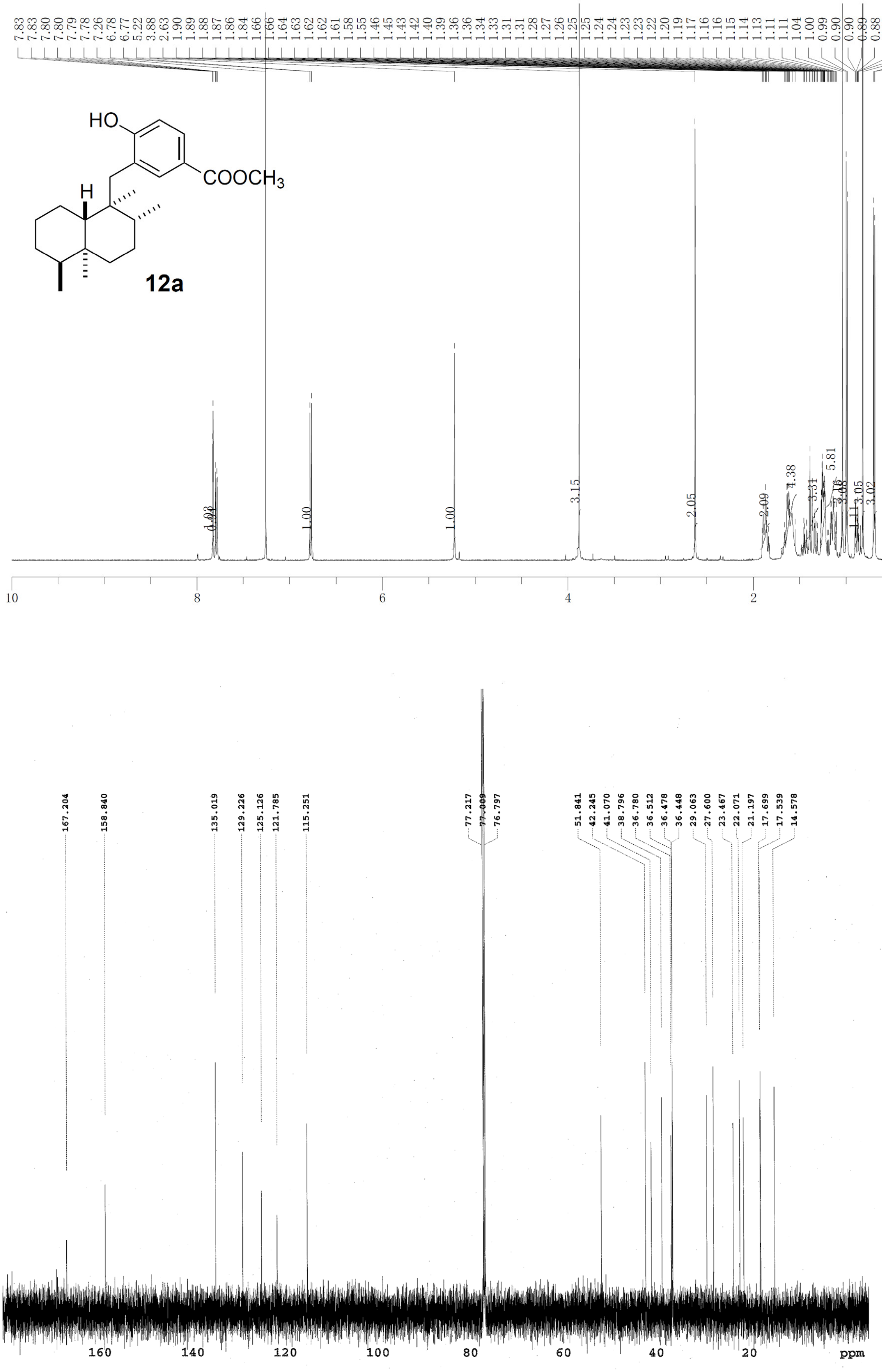

Figure S9. ^{1}H- and ^{13}C-NMR spectra of compound **12a**.

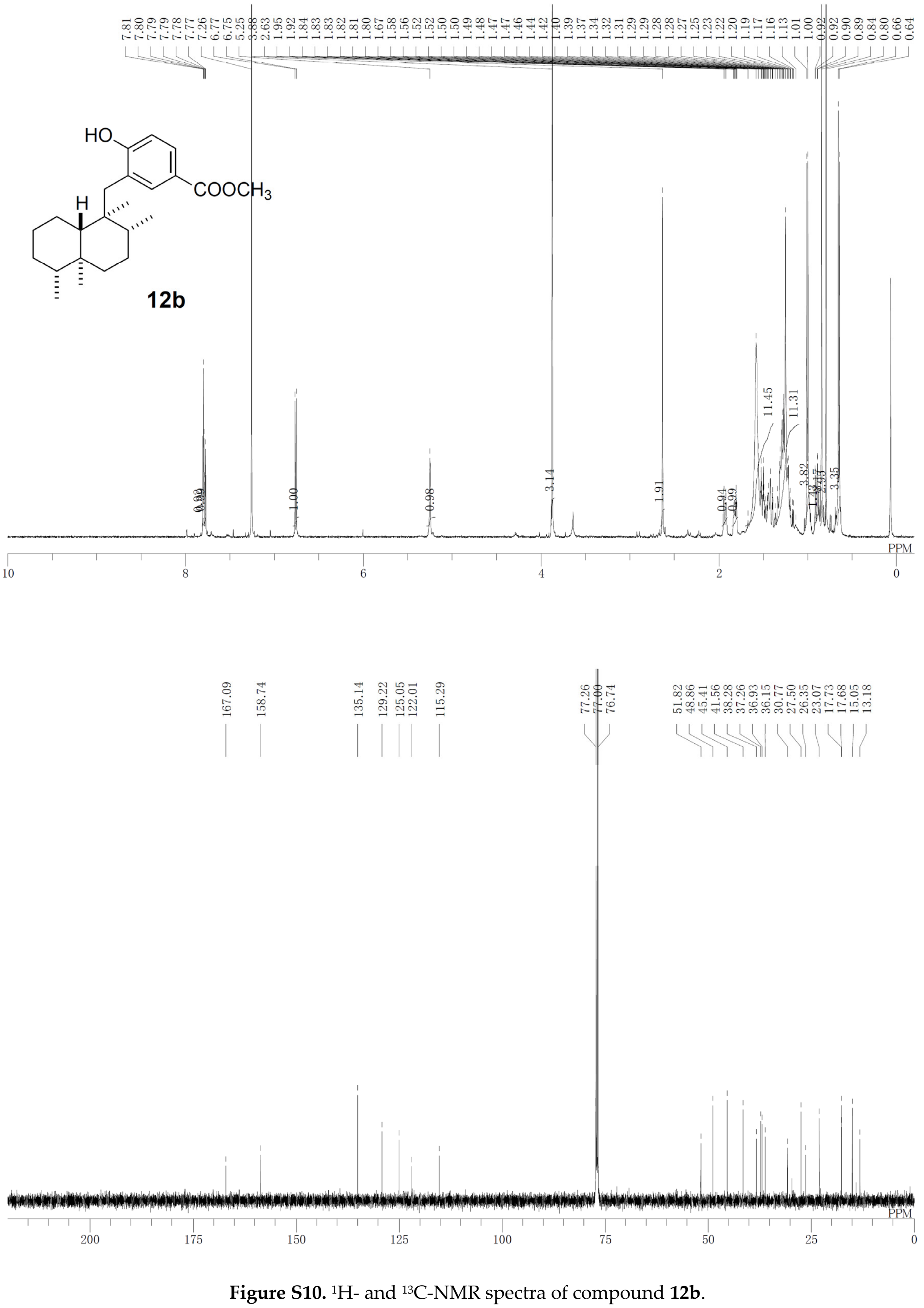

Figure S10. ^{1}H- and ^{13}C-NMR spectra of compound **12b**.

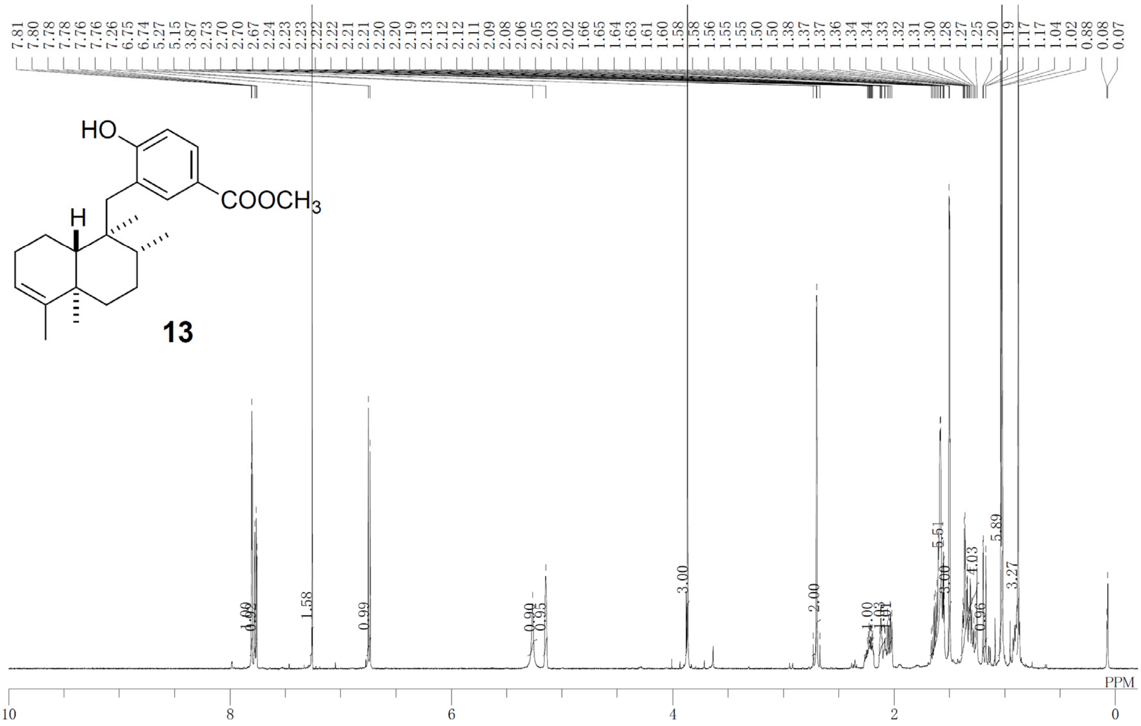

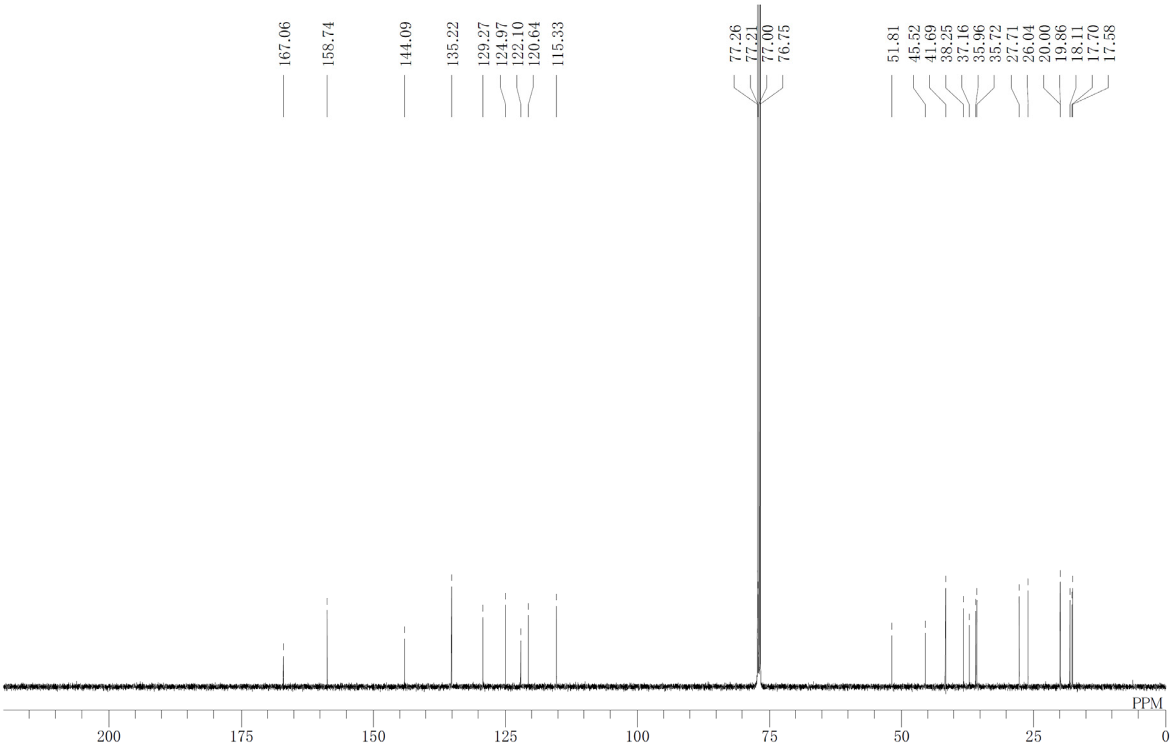

Figure S11. ^{1}H- and ^{13}C-NMR spectra of compound **13**.

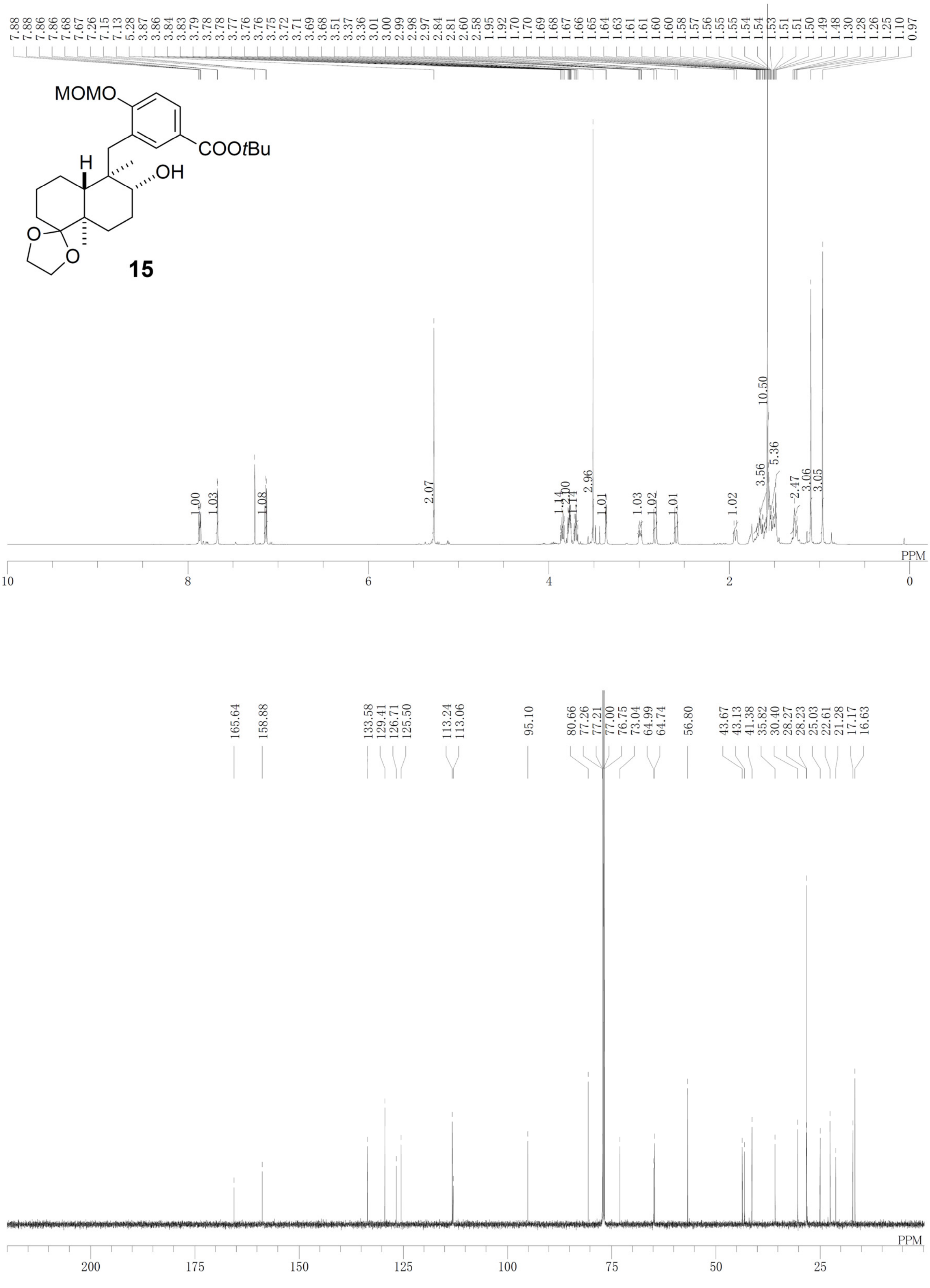

Figure S12. ^{1}H- and ^{13}C-NMR spectra of compound **15**.

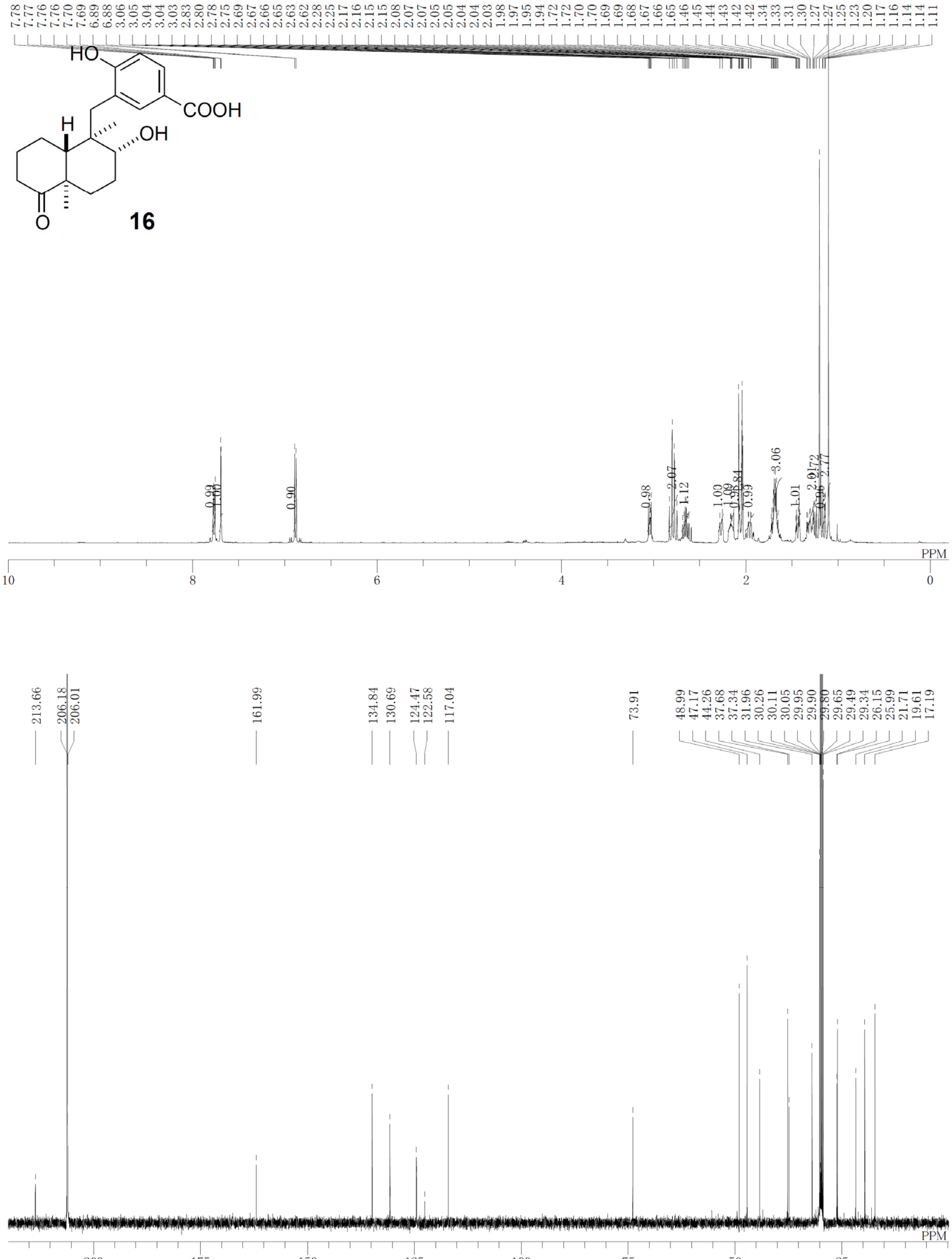

Figure S13. ^{1}H- and ^{13}C-NMR spectra of compound **16**.

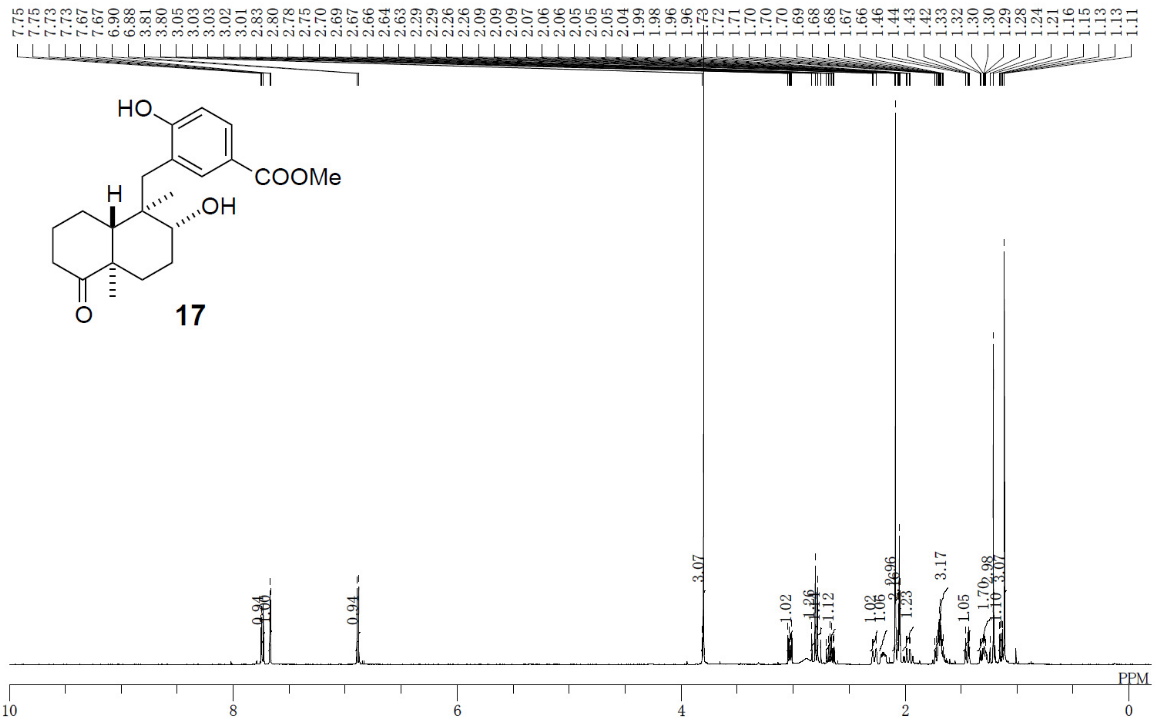

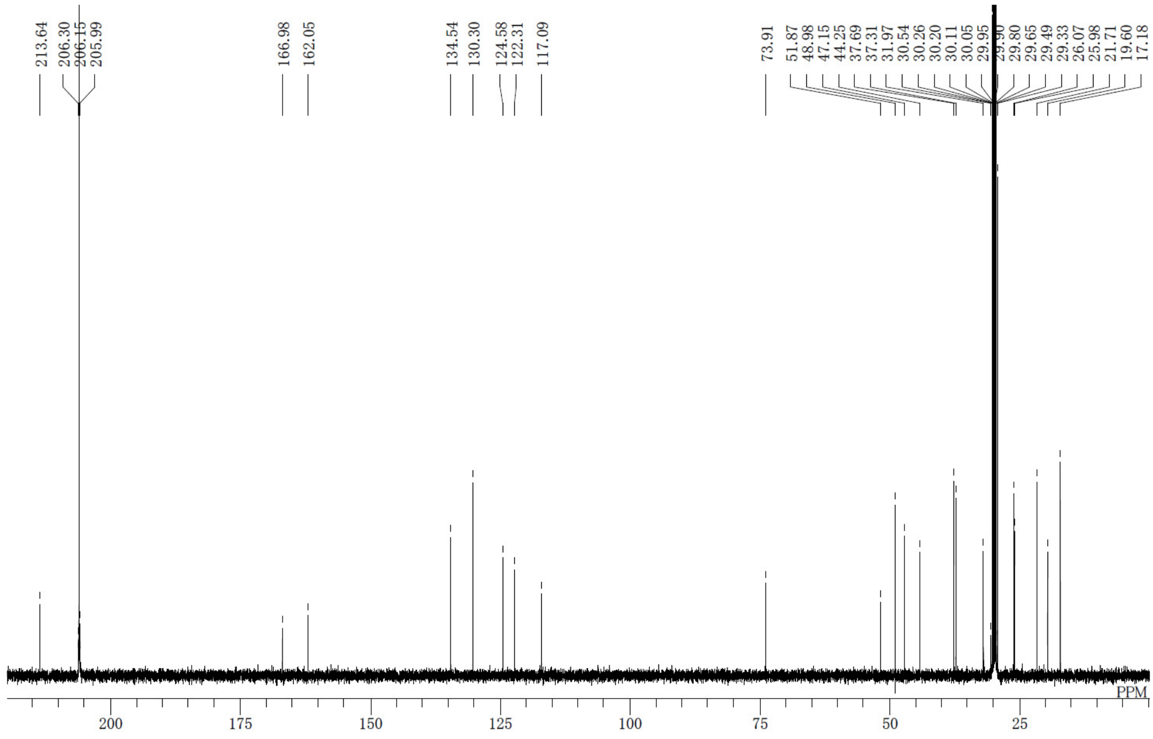

Figure S14. ^{1}H- and ^{13}C-NMR spectra of compound **17**.

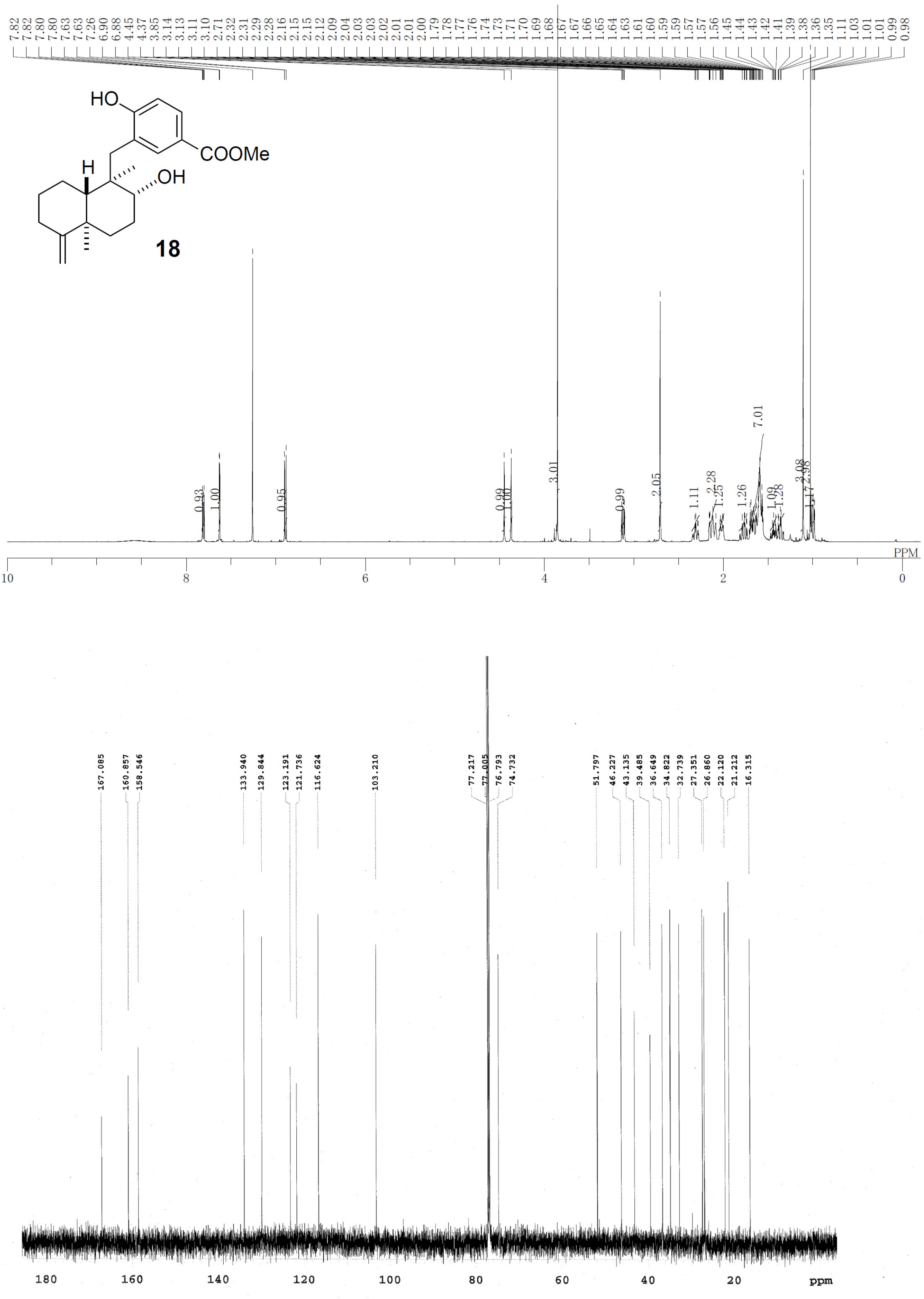

Figure S15. ^{1}H- and ^{13}C-NMR spectra of compound **18**.

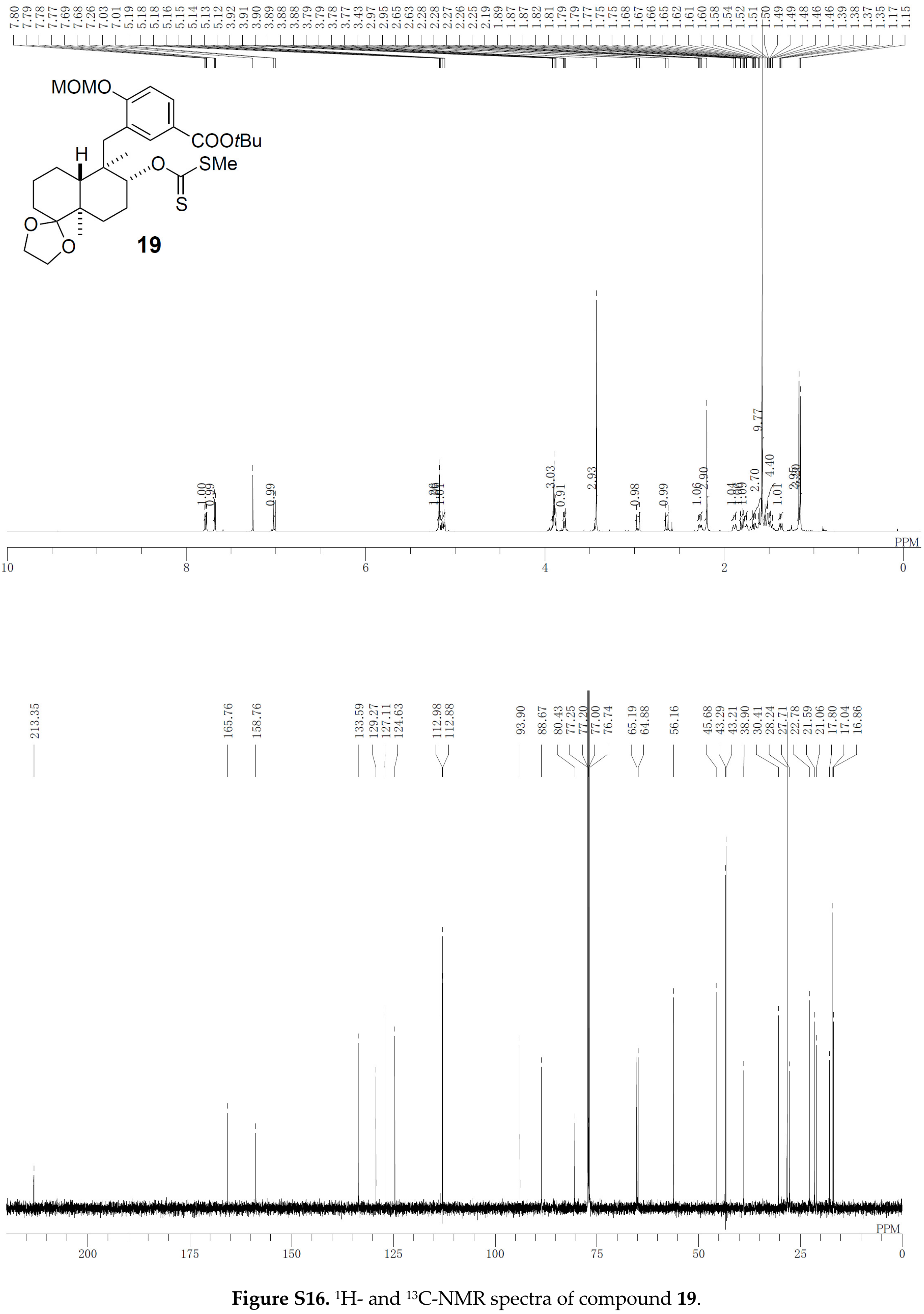

Figure S16. ^{1}H- and ^{13}C-NMR spectra of compound **19**.

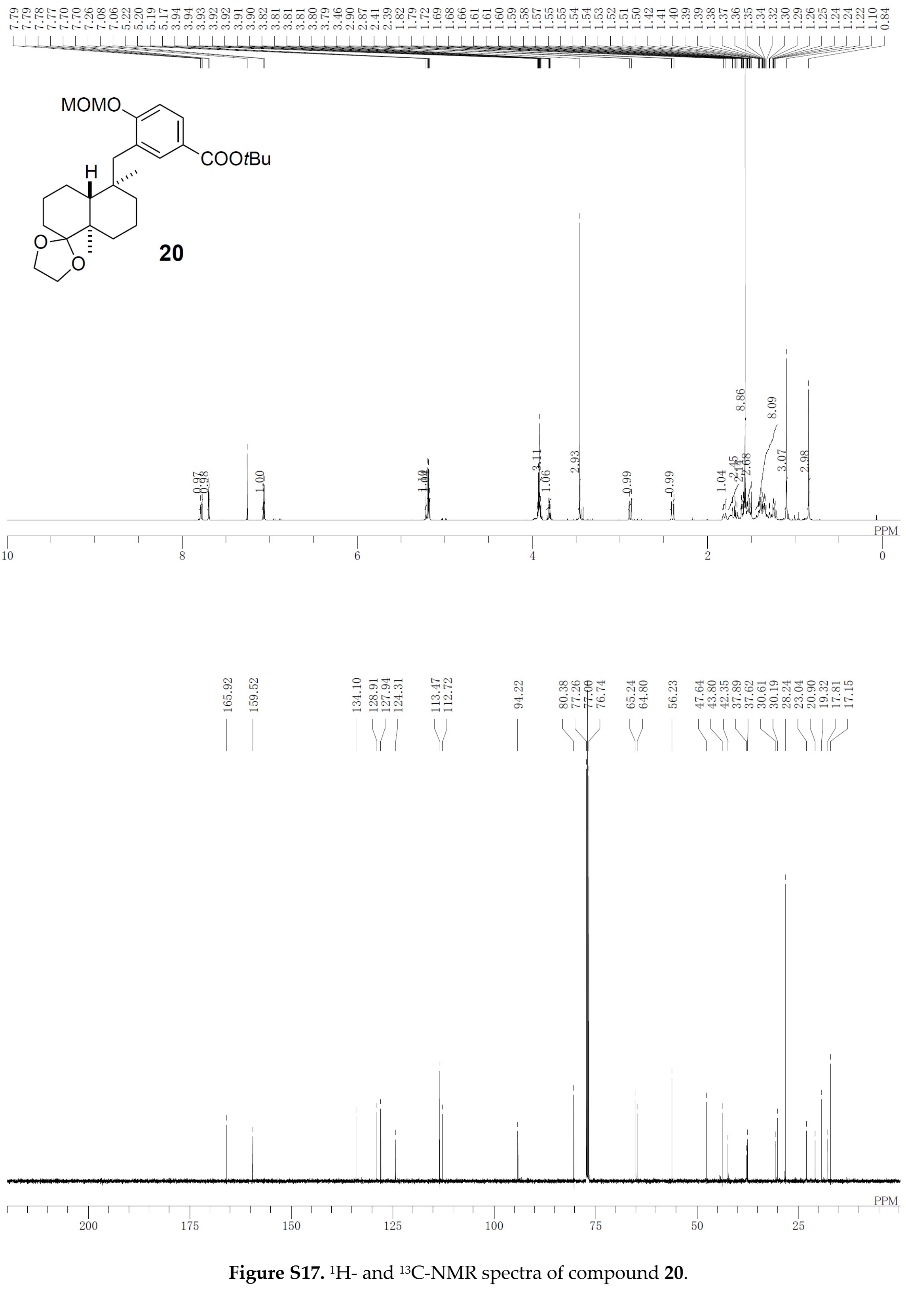

Figure S17. ^{1}H- and ^{13}C-NMR spectra of compound **20**.

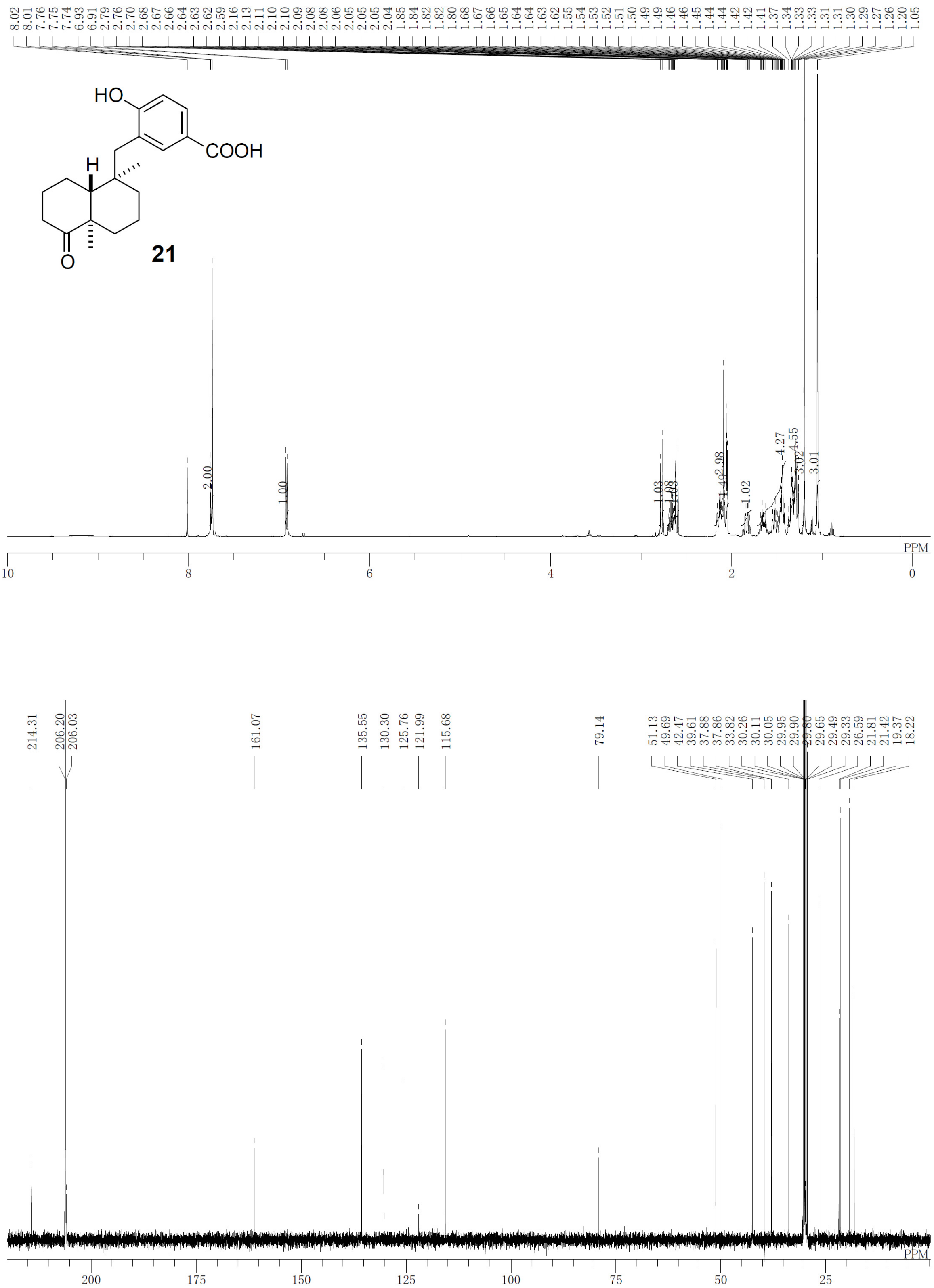

Figure S18. ^{1}H- and ^{13}C-NMR spectra of compound **21**.

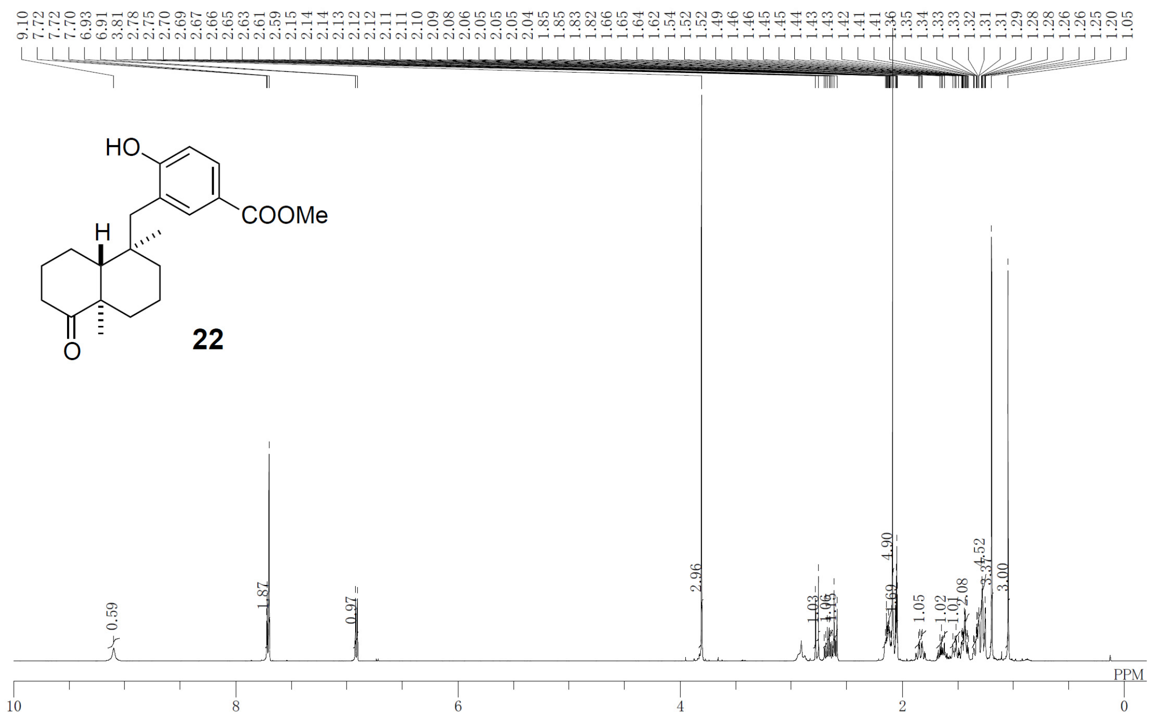

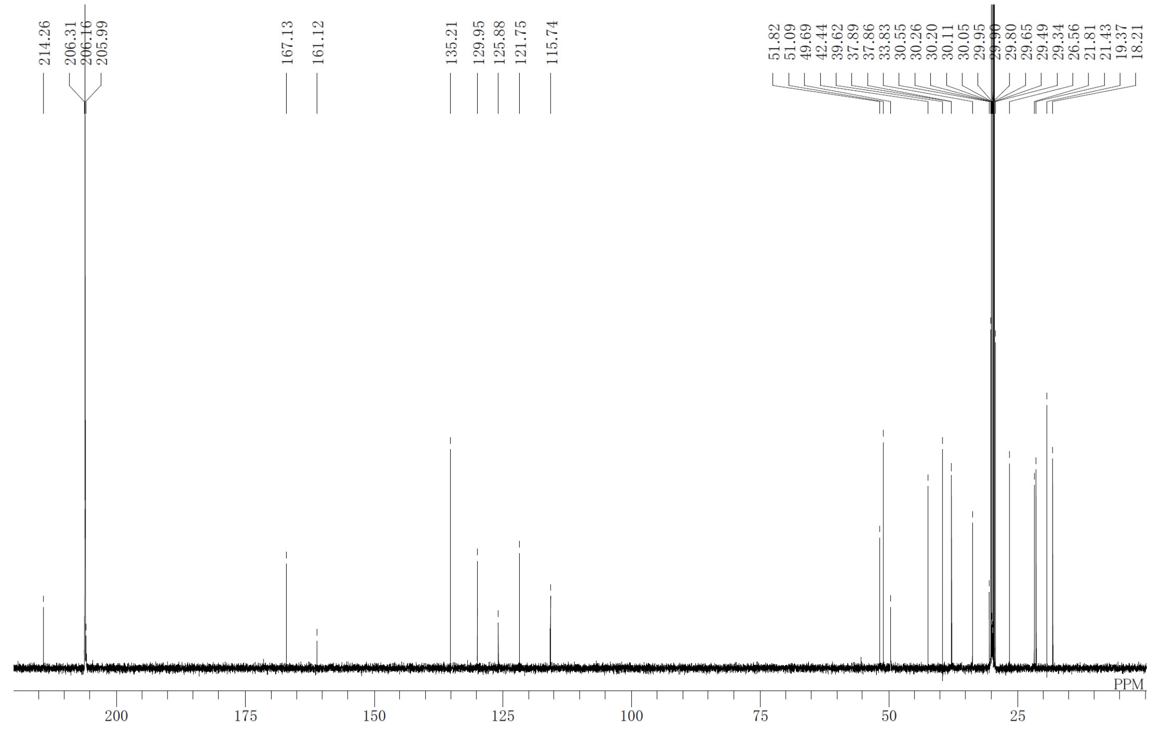

Figure S19. ^1H- and ^{13}C-NMR spectra of compound **22**.

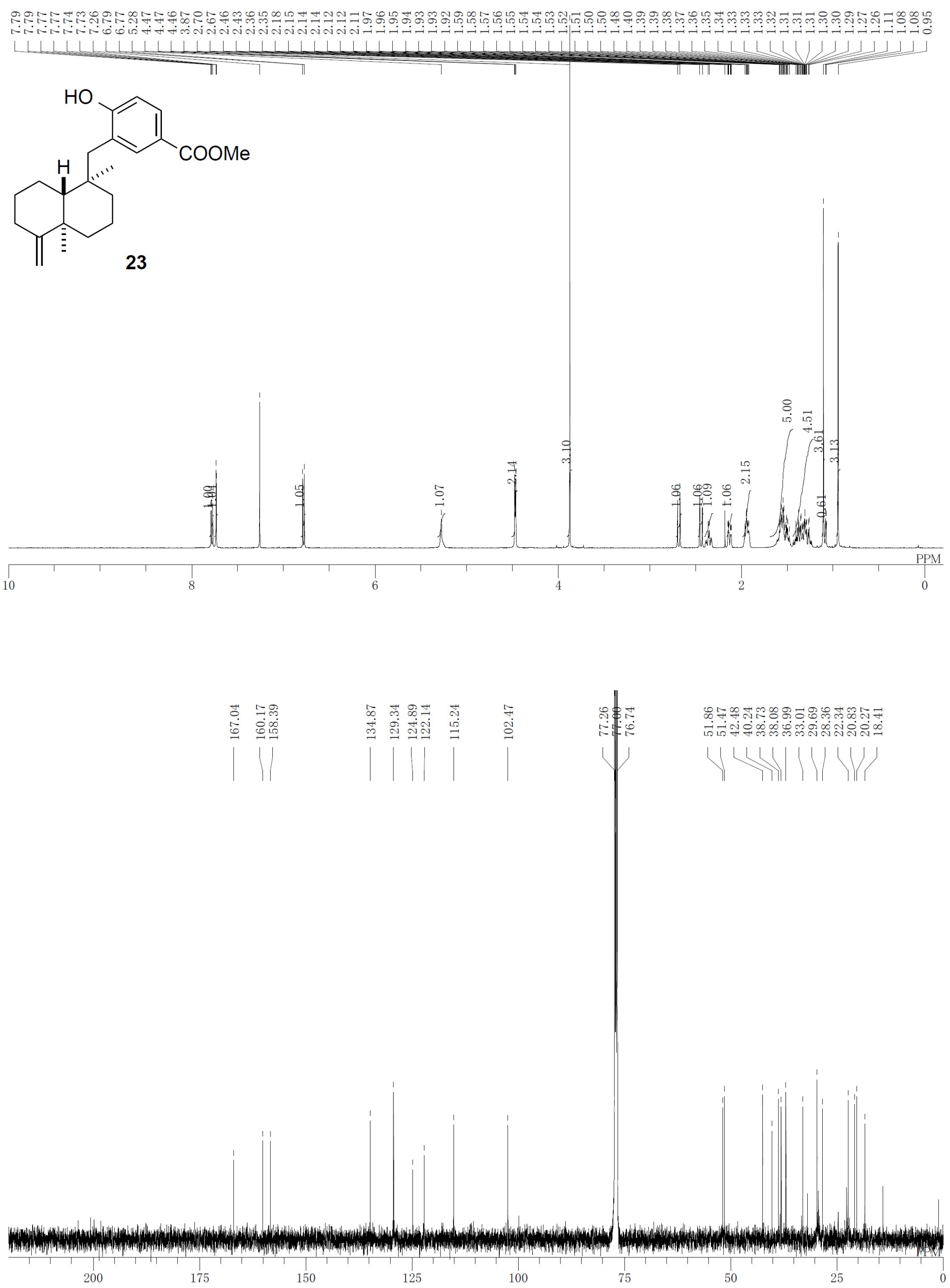

Figure S20. ^{1}H- and ^{13}C-NMR spectra of compound **23**.
